# State–space mark–recapture estimates reveal a recent decline in abundance of North Atlantic right whales

**DOI:** 10.1002/ece3.3406

**Published:** 2017-09-18

**Authors:** Richard M. Pace, Peter J. Corkeron, Scott D. Kraus

**Affiliations:** ^1^ Northeast Fisheries Science Center Woods Hole MA USA; ^2^ New England Aquarium Boston MA USA

**Keywords:** Bayesian mark–recapture, *Eubalaena glacialis*, open population abundance, recovery, survival

## Abstract

North Atlantic right whales (*Eubalaena glacialis* Müller 1776) present an interesting problem for abundance and trend estimation in marine wildlife conservation. They are long lived, individually identifiable, highly mobile, and one of the rarest of cetaceans. Individuals are annually resighted at different rates, primarily due to varying stay durations among several principal habitats within a large geographic range. To date, characterizations of abundance have been produced that use simple accounting procedures with differing assumptions about mortality. To better characterize changing abundance of North Atlantic right whales between 1990 and 2015, we adapted a state–space formulation with Jolly‐Seber assumptions about population entry (birth and immigration) to individual resighting histories and fit it using empirical Bayes methodology. This hierarchical model included accommodation for the effect of the substantial individual capture heterogeneity. Estimates from this approach were only slightly higher than published accounting procedures, except for the most recent years (when recapture rates had declined substantially). North Atlantic right whales' abundance increased at about 2.8% per annum from median point estimates of 270 individuals in 1990 to 483 in 2010, and then declined to 2015, when the final estimate was 458 individuals (95% credible intervals 444–471). The probability that the population's trajectory post‐2010 was a decline was estimated at 99.99%. Of special concern was the finding that reduced survival rates of adult females relative to adult males have produced diverging abundance trends between sexes. Despite constraints in recent years, both biological (whales' distribution changing) and logistical (fewer resources available to collect individual photo‐identifications), it is still possible to detect this relatively recent, small change in the population's trajectory. This is thanks to the massive dataset of individual North Atlantic right whale identifications accrued over the past three decades. Photo‐identification data provide biological information that allows more informed inference on the status of this species.

## 1 INTRODUCTION

Although measures of abundance are often deemed critical to development of wildlife conservation strategies, detecting trends in the abundance of populations of marine wildlife is a long‐recognized problem (Gerrodette, 1987). Broad‐scale surveys of oceanic species are especially problematic in this regard (Taylor, Martinez, Gerrodette, Barlow, & Hrovat, 2007), but even trends in the abundance of delphinids inhabiting small home ranges in inshore coastal waters can be difficult to determine (Parra, Corkeron, & Marsh, 2006; Wilson, Hammond, & Thompson, 1999). An exception to this general rule has been North Atlantic right whales (*Eubalaena glacialis* Müller 1776, Figure 1), for which an annual count, based on a near complete photographic census of the population, has been available for at least the past 25 years. During the period 1990–2011, this number had increased on average 2.8% per year to a minimum population count of 476 in 2011 (Waring, Josephson, Maze‐Foley, & Rosel, 2016).

**Figure 1:**
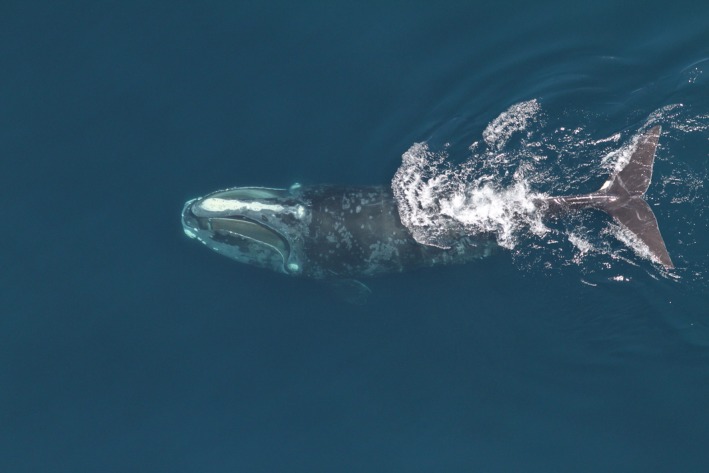
Overhead view of a feeding North Atlantic right whale, *Eubalaena glacialis*. Image collected under U.S. Marine Mammal Protection Act research permit number 17355. Photograph credit: National Oceanic and Atmospheric Administration/Northeast Fisheries Science Center/Christin Khan

How has it been possible to conduct a near complete census of a free‐ranging whale species? First, right whales are individually identifiable at an early age due to their unique callosity patterns (Hamilton, Knowlton, & Marx, 2007). North Atlantic right whales are designated as an endangered species under U.S. law, and most of the population spends a substantial amount of time in U.S. waters. They are subject to human‐caused mortality due to commercial fishing and shipping (van der Hoop et al., 2013; Knowlton et al., Conn & Silber 2013). Nonprofit science organizations, university researchers, and U.S. government agencies (state and federal) have pooled substantial boat and aerial survey efforts, to photographically identify individual whales, collect genetic samples, document calving and mortality events, assess health status, and collect evidence of entanglement in fishing gear through the North Atlantic Right Whale Consortium (Hamilton et al., 2007; Knowlton, Hamilton, Marx, Pettis, & Kraus, 2012; Rolland et al., 2016; Frasier, McLeod, Gillet, Brown, & White, 2007, http://www.narwc.org/). The resulting accumulation of individual resighting records forms the basis of an annual assessment of population status of North Atlantic right whales conducted by the U.S. National Marine Fisheries Service (NMFS). Because of their small population size, legal status, and efforts to mitigate human‐caused mortality, development of a regular, accurate evaluation of right whale abundance is essential to inform attempts to mitigate anthropogenic impacts.

North Atlantic right whales pose an interesting challenge for abundance estimation. Individuals can range from Florida to the Gulf of St. Lawrence and beyond, occasionally as far as northern Norway (Jacobsen, Marx, & Øien, 2004). Over the course of a year, there is nowhere among their favored habitats where all right whales are present at one time (Brillant, Vanderlaan, Rangeley, & Taggart, 2015; Brown, Kraus, Slay, & Garrison, 2007). However, their regular seasonal use of well‐known habitats in inshore waters has made the field effort and regular documentation possible. In particular, substantial aerial survey effort, specifically for the photo‐identification of North Atlantic right whales, has occurred in their southern (Florida, Georgia) calving grounds during the winter calving season (Keller, Garrison, Baumstark, Ward‐Geiger, & Hines, 2012) and through the Gulf of Maine almost year‐round (Roberts et al., 2016). There is no equivalent field program dedicated to any other whale species internationally.

The ability to identify individual whales at an early age due to their unique callosity patterns, coupled with annual surveys, albeit with variable effort, of most whale habitats for more than 30 years, has generated an extensive individual sightings database of most animals in the western North Atlantic right whale population. Previous studies have used these records to characterize the demographics of North Atlantic right whales. From those studies, it is apparent that, at a minimum, a priori consideration must be given to potential differences in survival rates and recapture probabilities among life stages, as these will affect the success of resighting individuals (Brown et al., 2001; Caswell, Fujiwara, & Brault, 1999; Fujiwara & Caswell, 2001). In this case, when sighting effort is effective and high, a trend in abundance is evident from a simple accounting procedure (Waring et al., 2016). During periods when sighting effort declines and is less effective, resulting in a decline in the probability of resighting individual animals, statistical approaches to estimate demographic parameters must be cognizant of these sources of heterogeneity.

However, since 2011, there have been changes in the distribution of North Atlantic right whales. For example, North Atlantic right whales have occurred in only very low numbers in the Bay of Fundy since 2011 (SDK, unpubl. data). The processes driving this change are unclear. One result of the changes in both whales' distribution, and in survey effort, has been that the likelihood of detecting each individual North Atlantic right whale each year has declined (Waring et al., 2016), thereby reducing the reliability of the previous, census‐based estimate of their abundance.

The open population models developed by Jolly (1965) and Seber (1965) were an important step in estimating abundance parameters from the periodic recapture of marked individuals because they allowed both recruitment and loss to occur between periods of recapture. If one can, on multiple occasions, randomly sample members of the study population and track the capture histories of all individuals caught at least once, then these data provide the opportunity to estimate both abundance and survival rates. Using open Jolly‐Seber mark–resight/recapture (MRR) models to estimate abundance is uncommon in ecology, because the resultant estimates of N are prone to bias resulting from capture heterogeneity (for example, see Nichols, Hines, & Pollock, 1984). Recent developments of open MRR models allowed for increased structural complexity of the models (see Williams, Nichols, & Conroy, 2002 for a review) to better match the complexity of biological and sampling processes they attempt to characterize. Most recent developments in MRR modeling, using Bayesian approaches to inference, have attempted to accommodate multiple sources of process and sampling noise and thereby reduce bias (Clark, Ferraz, Oguge, Hays, & DiCostanzo, 2005; Link & Barker, 2005).

Declines in abundance are an accepted indicator of concern for threatened and endangered species (IUCN 2012). When trends in absolute abundance can be developed on a regular and timely schedule, a robust picture of a species' status may emerge, and when combined with other demographic measures may lead to the better targeting of conservation strategies. We developed a Bayesian implementation of an open population MRR model to produce estimates of abundance and survival rates of North Atlantic right whales. We use these, together with observed annual calving rates, to assess the status of this population. With these results, we then assess the value of the past, and ongoing, photo‐identification survey effort for North Atlantic right whales for assessing trends in abundance of this species.

## 2 METHODS

We used information developed from the catalog of sightings records of photographically identifiable right whales (Hamilton et al., 2007) to estimate annual abundance and class‐specific survival rates for western North Atlantic right whales during 1990–2015. Resighting histories of known individuals were used to estimate survival rates and abundance in a Bayesian, state–space formulation estimated using markov chain monte carlo (MCMC) simulation. Animals enter the study when a credible suite of photographs are taken that allow near error free recognition (Frasier, Hamilton, Brown, Kraus, & White, 2009). While others have used catalog data collected since 1980 to characterize right whale survival (Fujiwara & Caswell, 2001, Robbins, Knowlton & Landry,2015), we were concerned that during the early development of the catalog (1980–89), there were retrospective recaptures hidden within the histories of individuals. A retrospective recapture occurs when adequate identifying features are fully photographed in one year allow a researcher to identify a previously captured but inadequately photographed animal from archived images and thereby increase the known life span within the capture history. The presence of retrospective recaptures would inflate survival rates, because animals poorly photographed that die before they are seen again cannot be retrospectively recaptured. In addition, prior to 1990, surveys of the calving area were limited, which greatly reduced the likelihood of capturing some individuals. To avoid the influence of retrospective recaptures and the effect of reduced early survey effort in the calving area, we limited the estimation of parameters to the period 1990–2015. However, information about animals identified prior to 1990 was used to inform initial values and the known states and age covariates during the study period.

### 2.1 The data

We acquired data on 61,178 sightings of cataloged individual North Atlantic right whales extracted on 25 October 2016 from a database curated by the New England Aquarium (NEAq, Boston, Massachusetts, USA). Identifications of individual whales were provided by NEAq personnel and based primarily on photo‐identification using natural markings (Hamilton et al., 2007; Kraus et al., 1986) and supplemented with genetic markers (Frasier et al., 2007). We considered the survey year to be 1 December‐30 November because late fall (October and November) represents a period of very few sightings over the study period and because December marks the beginning of the right whale calving season. That is, the “1990” year starts on 1 December 1989 and ends on 30 November 1990. Capture histories were built by compressing sighting records of individual whales during a year (often multiple sightings of the same individual in multiple geographic areas throughout the year) into a single binary observation (seen or not seen). Thus, the occurrence of each whale during the 26 annual sample periods during 1990–2015 constituted a capture history, and together these histories became the principal data used to estimate abundance and survival.

Capture histories were used to develop a state matrix. For each whale, any capture interval for which it was known to be alive was coded as state_it_ = 2, where the subscript *i* refers to the individual whale, and t refers to the year. Any period during and after which a whale was discovered dead was coded as state_it_ = 3. Any period prior to the birth year of a known‐age whale was coded as not yet entered (state_it_ = 1). All other values in the state matrix were coded as unknown (NA). Known states were frequently informed by information gained prior to 1990 if an animal was known to be alive prior to the first time it was seen during 1990–2015. (Example, an animal seen in 1989 but not seen again until 1992 was given states of 2 for 1990 and 1991 as well as any year up to the last year that it was seen). If an animal was of unknown age when first seen after 1990, states in the data matrix prior to the year first seen were treated as unknown (NA). In addition to the primary data, the known states were informed by a sighting records posted online (http://rwcatalog.neaq.org) after 25 October 2016 for evidence of sightings after 30 November 2015.

To further inform the modeling process, we also used other information associated with the resighting of individual whales, including known birth and death years, sex, and age. To accommodate the possible effect of differential survival among the youngest age groups, we categorized animals to one of 6 age classes, 0, 1, 2, 3, 4, and 5+ (animals 5 and older). For purposes of estimating age‐related survival, animals of unknown age at entry were treated as though they were age 5+.

### 2.2 Analysis

To estimate abundance and survival of North Atlantic right whales, we followed Kéry and Schaub's (2011) and Royle and Dorazio's (2012) outlines of a multistate formulation for the estimation of a J‐S model of MRR data in a Bayesian framework. Expanding upon that approach, we separated the likelihoods associated with state transition or biological process from that of the observation process. The biological states modeled were as follows: (i) not yet entered into the population, (ii) alive, and (iii) dead. The two observed states were seen or not seen. To account for the possibility that an animal might enter the population and yet never be seen, which is a necessary parameter for the derivation of abundance estimates, we augmented the capture histories (Royle & Dorazio, 2012). Data augmentation, as used in a Bayesian capture–recapture framework, is a modeling process to address the occurrence of unobserved individuals in a population of interest. Royle and Dorazio (2012) describe data augmentation of capture–recapture data in detail. In this instance, we allowed that as many 200 additional individual whales may have entered the population but were never captured during our study period. The number actually estimated to have entered but were never seen results from estimating the probability of entry which is one of the model parameters.

The open population mark–recapture model of Seber (1965) made assumptions of capture and survival probability homogeneity among individuals, which is often extended to groups in more complex models (Williams et al., 2002). Most long‐lived mammals show variation in survival rates according to sex and age (Caughley, 1966). In addition, Cormack‐Jolly‐Seber (CJS) models fit to earlier subsets of North Atlantic right whale catalog data suggested that knowledge of sex and age/stage should be used to reduce capture and survival heterogeneity (Caswell et al., 1999; Fujiwara & Caswell, 2001; RMP unpublished data). Finally, abundant evidence exists demonstrating that (i) effort and success of resighting whales have varied over time (Hamilton et al., 2007), (ii) estimated survival of whales has varied with time (Fujiwara & Caswell, 2001), and (iii) individual capture probabilities are heterogeneous due to differential use among habitats by individual whales and by different demographic groups, (Brown et al., 2001).

To accommodate heterogeneity in capture and survival rates, we incorporated linear relationships (Lebreton, Burnham, Clobert, & Anderson, 1992) to the logit of survival and capture probabilities. Survival probability was modeled as:

$$\text{Logit}\left(\phi_{i,t}\right)=\beta_{1}+\beta_{2}*\left({\text{sex}}_{i}\right)*{\text{Adult}}_{i,t}+\beta_{3}*{\text{Age}}_{i,t}+\varepsilon_{t}$$

where ϕ_*i,t*_ is survival of probability of the *i*th individual for the *t*th interval, β_1_ is the intercept whose value in the logit is the mean of calf survival, β_2_ is the added effect of being a female > 4 years old on survival, sex_*i*_ is a data value of 1 for female, 0 for male, and NA for unknown, Adult_*i,t*_ is a data value of 1 if the *i*th animal is classed as age >4 in the *t*th interval, β_3_ is the linear effect of age, Age_*i,t*_ is a data value ranging from 0 to 5 for the *i*th individual at time interval *t*, ε_*t*_ is the random effect of year on survival.

Similarly, we modeled capture probability as:

$$\text{Logit}\left(p_{i,t}\right)=\alpha_{1}*\left({\text{sex}}_{i}\right)+\alpha_{2}*\left(1-{\text{sex}}_{i}\right)+{\text{Time}}_{t}+\zeta_{i}$$

where α_1_ was the intercept and hence the effect of being a female on capture probability, α_2_ was the added effect of being a male on capture probability, Time_*t*_ was the added effect of the year *t* (a factor) on average capture probability with Time_*t*_ = 0 for *t* = 1990, ζ_*i*_ was the random effect of the *i*th individual on capture probability.

For estimation, we assigned vague priors on all linear terms in the logit except the random coefficients ε_*t*_ and ζ_*i*_, as uniform (−10, 10). Random coefficients ε_*t*_ and ζ_*i*_ were given normal (0, δ) and normal (0, σ) priors, respectively. Standard deviation terms δ and σ were given vague priors of uniform (0.001, 10). The probability of entry into the population, γ_*t*_, was allowed to vary among time intervals, and each γ_*t*_ was assigned a uniform (0, 1) prior. Transitions among states (not yet entered, alive, or dead) were modeled as a discrete categorical random variable dependent on the prior state according to the following probabilities (common table which shows the current state in the first column and the probabilities to transition to the other states in the following columns):

**Table:** 

	Not entered	Alive	Dead
Not entered	1 − γ_*t*_	γ_*t*_	0
Alive	0	ϕ_*i,t*_	1 − ϕ_*i,t*_
Dead	0	0	1

The observed data (seen or not seen) were considered dependent on the animal's state and were modeled as Bernoulli (p[s]) according to the following:

**Table:** 

State	Seen	Not Seen
Not entered	0	1
Alive	*p*_*i,t*_	1 − *p*_*i,t*_
Dead	0	1

Finally, missing data on the sex of individual whales were modeled as Bernoulli (ρ), where ρ was given a somewhat informative beta (5, 5). Using the above structure, data were modeled using program JAGS (Version 4.2) MCMC simulator (Plummer, 2003) accessed via R statistical program (R Development Core Team 2012) and package run.jags (Version 2.0.2‐8, Denwood, 2016). When dealing with model parameters in all simulation exercises, we provided random starting values from within the range of the prior for that parameter. We provided initial values for unknown states (state.init_it_) which were state.init_it_ = 1 prior to the first year seen and state.init_it_ = 3 after the last year seen. Unknown sexes were assigned a Bernoulli (0.5) random initial value. We used an adaptation + burn in phase of 5,000 iterations and sample size of 20,000 iterations for estimation. JAGS code for the primary model is provided in a Supporting Information. In all cases, to determine when the algorithms had converged, we used three chains and computed the Gelman–Rubin convergence statistic, which we required to be <1.1 for all model parameters (Gelman & Rubuin, 1992). For starting values in the known states as data instance, missing values for all known data quantities were submitted, including a value of 3 for all instances after the last year seen when not known to be dead, and a value of 1 for all animals in the augmentation set of capture histories. Covariates concomitant with capture histories in the data augmentation set were unknown for sex and age = 5 and adult = 1 adult for age class.

As further support for our model choice and lack of sensitivity to assigning latent ages to the 5+ class, we conducted a simulation study which is described in the Supporting Information .

### 2.3 Minimum number alive

Because right whales are long lived and because this population is surveyed so heavily, a relatively straight forward accounting procedure has been used to characterize their abundance. As an accounting exercise separate from the statistical model described above, minimum number alive (MNA) was calculated as the count of all animals known to be alive in a year, because they were either seen in that year or seen in the years before and after that year. MNA will be less than or equal to the actual population size, because it misses animals alive but not yet cataloged and animals still alive after the last year in which they were seen. However, the combination of high annual capture rates and high survival among right whales should make this bias small but tending to increase toward the end of the study period. As a comparative measure, we calculated MNA for each year in 1990–2015 and plotted those values together with posterior medians from the Bayesian hierarchical model.

### 2.4 Fecundity

Maintained along with the sightings histories of individuals are annual calf production data (Kraus, Pace, & Frasier, 2007). The detection of a calf occurs through photo‐identification of an adult female being accompanied by a calf in the wintering area. The area is heavily surveyed and the rate of entry of animals of unknown age entries of individuals into the photo‐identification catalog indicates that calves have been rarely missed since 1990. We calculated an annual per capita productivity index (API) as:

$$\begin{array}{cc} \text{API}\;=\; & (\text{number of calves detected})/\\ & \left(\text{estimated population size for each time period}\right) \end{array}$$

We plotted these values over time to look for patterns that, together with estimates of survival, may help explain any trends in population size or crude growth rate. We calculated an annual crude population growth measure as:

$${\text{Growth}}_{t}\;=\;N_{t}/N_{t-1}$$

where the values for *N*_*t*_ were taken as the median values among the MCMC chains. Using the idea of growth constructed thusly, a post hoc evaluation of periodic growth between period *t* and period *t*−*k* and associated uncertainty could be calculated as attributes of the posterior distribution of the calculated *N*_*t*_/*N*_*t*−*k*_ for each MCMC iteration.

## 3 RESULTS

This analysis included capture histories from 658 whales, including 280 females, 328 males, and 50 animals of unknown sex. Of the 658 individual whales seen during the study period, 247 were first seen prior to 1990. Of the remaining 411 whales, 101 were at least 1 but otherwise were of unknown age, and treated as though they were 5+ for purposes of survival estimation. The primary multistate Bayesian MRR model employed here had excellent convergence statistics as judged by the computed Gelman‐Rubin convergence statistics (All parameter estimates and associated MCMC attributes are available in the Supporting Information) and posterior distributions for all linear (in the logit space) parameters associated with time. Sex and age covariates contributed significantly (i.e., were distinct from zero) to estimates of survival and capture probability.

**Figure 2:**
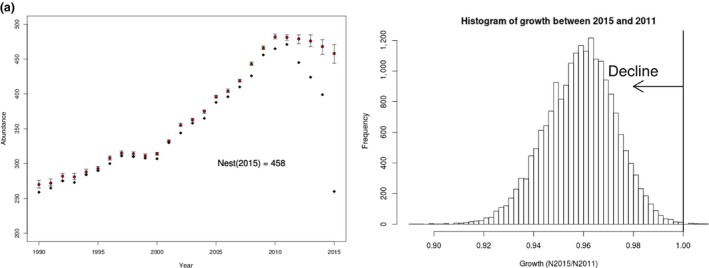
Abundance of North Atlantic right whales 1990–2015 as estimated from mark–resight data as calculated from two procedures. Black diamonds are counts of the minimum number of individuals seen alive (MNA) that year plus those seen before and after that year. Circles with error bars are posterior medians and associated 95% credible intervals from a Bayesian mark–recapture model allowing random fluctuation among years, age effects and adult female effects on survival, as well as sex and time effects and random effects of individual catchability on capture probabilities together with their 95% critical regions. Inset shows the posterior distribution of estimated growth of the North Atlantic right whale population between 2015 and 2010 measured as a ratio (N_2015_/N_2010_) of abundance estimates from Bayesian implementation of J‐S model. Almost all (99.99%) of the estimates of growth are below 1.0, indicating a population decline

Estimated abundance mimicked MNA values quite closely until the last few years of the study period, when as expected, estimated abundance did not drop precipitously as did MNA (Figure 2). In addition, lower bounds of 95% highly credible intervals were usually at or above MNA, a value previously used by NMFS to judge the status of the right whale population (Waring et al., 2016). Estimated population size showed relatively consistent slow growth during 1990–2010, with two small inflections prior to 2010. There was a likely one single‐year decline in median abundance during 1993, and a brief period (1997–2000) of no growth shortly thereafter. Our analysis estimated a 99.99% probability that the 2015 abundance represents a decline since 2010 (Figure 2, inset).

**Figure 3:**
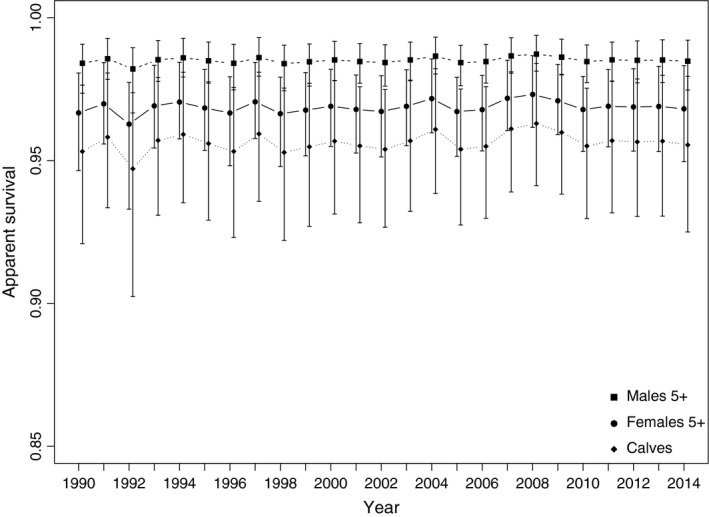
Estimated survival rates and associated 95% credible intervals of three classes of North Atlantic right whales 1990–2015 based on a Bayesian implementation of J‐S model allowing random fluctuation among years and using known states as data

Estimated survival rates from the model showed relatively minor random fluctuations in survival among years (Figure 3). The means of the estimated mean survival rates (Column 4 in Supporting Information  I) and mean of their estimated *SD* (Column 5 in Supporting Information I) were as follows: for males, 0.985 ± 0.0038; for females, 0.968 + 0.0073; and for calves 0.955 ± 0.0127.

**Figure 4:**
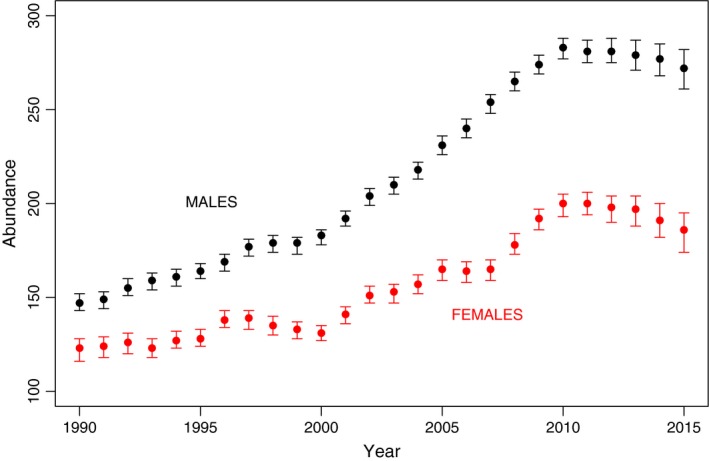
Median abundance and associated 95% credible intervals by sex of North Atlantic right whales 1990–2015 based on a Bayesian MRR model allowing random fluctuation among years for survival rates, treating capture rates as fixed effects over time, and using both observed and known states as data

Reduced survival of 5+ females compared to 5+ males has resulted in diverging trajectories in male and female abundance (Figure 4). In 1990, there were an estimated 142 males (95% credible intervals 143–152) and 123 (116–128) females (Figure 4, also see Supporting Information I), or 1.15 males per female. By 2015, model estimates indicate that the species comprised 272 (261–282) males and 186 (174–195) females, or 1.46 males per female. Figure 4 also shows that the previous inflections in the trend in the abundance of North Atlantic right whales were due to decreases in female abundance, not male abundance.

**Figure 5:**
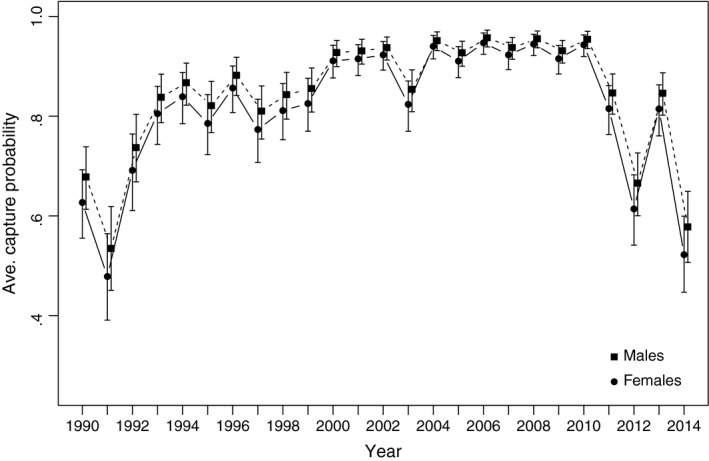
Estimated recapture probability and associated 95% credible intervals of North Atlantic right whales 1990–2015 based on a Bayesian MRR model allowing random fluctuation among years for survival rates, treating capture rates as fixed effects over time, and using both observed and known states as data

In contrast to small amounts of variability apparent in estimated survival rates, estimated mean capture probability was modest early, rose to about 90% until 2011, and dropped off to between 65% and 80% during 2012–2015 (Figure 5).

**Figure 6:**
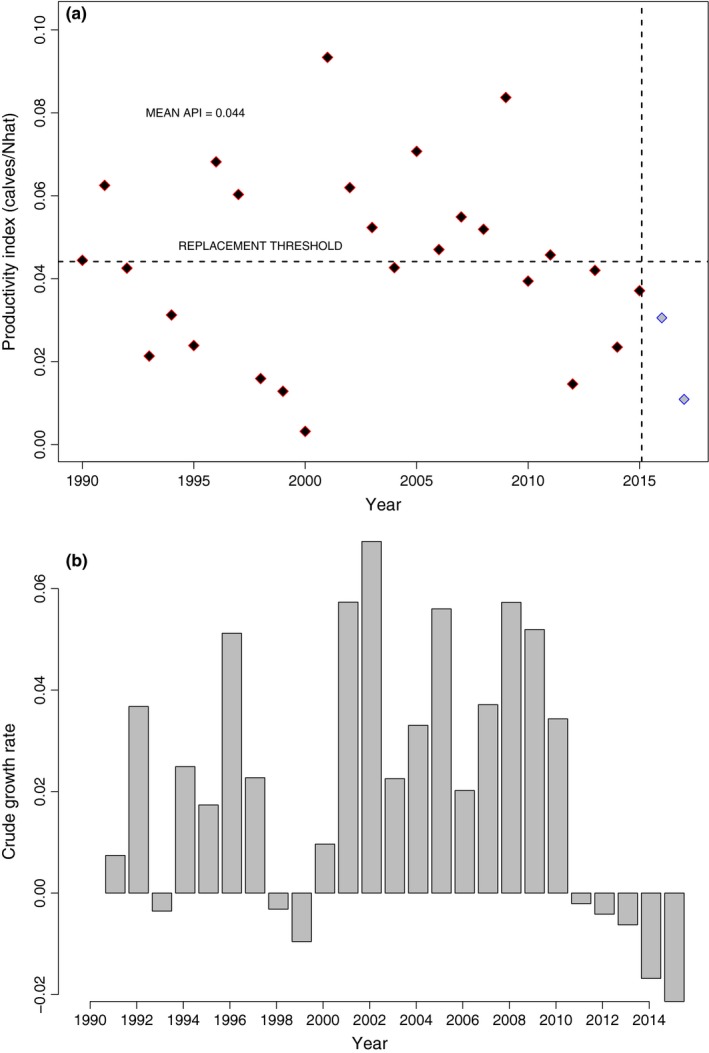
Annual productivity index (a) for North Atlantic right whales calculated as the number of detected calves/(median of posterior distribution of Estimated population size) and (b) crude growth (*N*_*t*+1_/*N*_*t*_) rate based on model medians. Note that the last two points in plot (a) assume the 2015 population size for the calculation of API

Calf production, when viewed as a per capita output, varied considerably during the study period (Figure 6) averaging 4.4% and ranging from 0.3% to 9.5%. Three periods of very low per capita production (1993–95, 1998–2000, and 2012–2015) coincided with no or negative growth (Figure 6b) during 1992–93, 1997–2000, and 2011–2015 which is evident when abundance estimates are viewed as a time series (Figure 1).

## 4 DISCUSSION

### 4.1 How many North Atlantic right whales?

Although there is a substantial literature on the biology of North Atlantic right whales (Kraus & Rolland, 2007), this is the first published estimate of their abundance that has been derived using a statistical model. The point estimate of abundance of North Atlantic right whales increased at approximately 2.8% per year over the first 21 years of the time series, from 270 in 1990 to 482 in 2010, after which this increase has leveled out, and declined to 458 (95% credible intervals [444, 471]) in 2015. Of particular concern is the divergent trends in the abundance of male and female North Atlantic right whales. In both 2010 and 2011, there were estimated to be 200 (combined 95% credible intervals 193–206) females in the species, declining to 186 (174–195) in 2015. Males declined from a peak of 283 (277–288) in 2010, to 272 in 2015 (261–282).

Examining the credibility intervals for all estimates from during 2010–2015 makes it difficult to determine whether the apparent decline is real. However, examination of the posterior values of growth from 2010 to 2015 (*N*_2015_/*N*_2010_) strongly suggests a decline (Figure 2, inset). The probability that growth < 1 (i.e., a decline) from 2010 to 2015 was 99.99%. Median growth from 2010 to 2015 was 0.950 (95% credible interval: 0.925, 0.971), suggesting a decline in abundance of 5% overall, or just under 1% per year over that period. Broken down by gender, males declined just under 4%, and females declined approximately 7%.

Prior to our work, two series of estimates, both based on enumerating known individuals, have been available. One, the MNA described above, has been used to inform NMFS stock assessments (Waring et al. 2016) and was structured to meet a specific legal requirement under the U.S. Marine Mammal Protection Act (MMPA) for all marine mammal stocks under U.S. jurisdiction. Because all whales are not detected each year, the last few values in a time series suffer from the increasing probability of assuming a whale is dead when it is still alive, a condition which worsens as annual capture probabilities decline.

A second estimate is produced each year by the North Atlantic Right Whale Consortium (http://www.narwc.org/) from an approach developed by the New England Aquarium (Kraus, Hamilton, Kenney, Knowlton, & Slay, 2001; Kraus et al. 2016). It differs from the MNA above in that an identified individual whale is assumed to be alive until seen dead or not observed for six years after the last year seen. The Consortium is explicit that this is not a true population estimate (e.g., NARWC 2015), although it tends to be treated as one. By assuming a mortality schedule for undetected whales at the end of the time series, the consortium approach buffers the declining probability of detecting a whale that is still alive toward the end of the time series but has the potential of positive bias in estimated abundance if the assumed mortality schedule does not match the true mortality schedule. While the effect of declining capture success would produce MNA values likely construed as a decline in abundance, the Consortium approach has the potential to lag in its detection of a true decline at the end of a time series.

Changes in habitat use patterns among North Atlantic right whales in recent years coupled with reduced resources for surveying the more distant areas of their known range mean that resighting rates of individual whales have been declining in the past few years (Figure 5). Given these data issues, and the well‐known problems with bias at the end of a time series of MNA estimates (Pocock, Frantz, Cowan, White, & Searle, 2004), we developed this statistical framework for estimating North Atlantic right whales' abundance. The new abundance estimates track the MNA closely through most of the time series, and the new point estimates always lie above the MNA (as is mathematically appropriate). Most significantly, the structure of our model provides confidence that the observed lack of increase in North Atlantic right whales' abundance since 2011 is not due to reduced detection of whales in recent years, rather it reflects a true change in trend.

### 4.2 Survival and reproductive rates

Between 1990 and 2015, annual survival rates of male North Atlantic right whales over four years of age fluctuated little, at around 0.98. Annual survival rates for females 5+ were lower, at around 0.97, leading to the current situation where there are substantially more males than females in the population (Figure 4). Assuming a linear (in the logit space) change in survival from 0 to the 5+ class contributed significantly to reducing deviance. The resulting coefficient had a posterior median of 0.296 (95% h.c.r = [0.187, 0.404]), which due to the direct relationship between coefficient and odds ratios (Hosmer & Lemeshow, 1989), can be interpreted as the odds for survival increasing at about 30%/year of age for the first 5 years of life, but likely biased somewhat low due to pooling animals of unknown age into the 5+ class. This relationship produced estimated survival rates of calves that ranged from 0.894 to 0.922, with animals ages 1–4 having estimated survival rates intermediate between calves and 5+ year old males.

Estimated calf survival is likely biased high for two reasons. First, constraining heterogeneity in survival among age classes to a linear (in the logit space) relationship might have proved limiting, but more importantly, most known‐age animals enter the catalog (i.e., develop callosity patterns that make them identifiable) at about 6 months. Thus, survival estimates for that class represent about ½ a year. Ecologically, the viability of younger right whale age classes (1–4), although quite high, indicates that experience matters in this long‐lived whale species living in an anthropogenically perturbed environment. These estimated survival rates are not directly comparable to previous estimates (Caswell et al., 1999; Fujiwara & Caswell, 2001) as the models were constructed very differently and cover different time periods. While these estimated survival rates for animals 5+ appear higher than previous estimates, we believe this is due to known mortality information used in these models and not used in Cormack–Jolly–Seber formulations similar to that used by Caswell et al. (1999) or the multistage model of Fujiwara and Caswell (2001).

Annual per capita calving rates averaged only 4.4% and showed substantial annual variability. These rates are low and variable when compared with calving rates of congeneric Southern right whales, *E. australis*, (Best, Brandão, & Butterworth, 2001; Carroll et al., 2013), for which the API would be roughly 8%, assuming total mortality of 2% and the observed population growth rate of 6%. Periods of poor calving in the mid and late 1990s and 2012–2015 are evident (Figure 6a). Assuming the same population size as in 2015, the API in 2016 and 2017 has also likely been less than needed for replacement of dying whales (Figure 6a), which suggests that abundance will continue to decline through 2017. Calf production in North Atlantic right whales has been linked to right whale health (Rolland et al., 2016), oceanographic processes (Meyer‐Gutbrod, Greene, Sullivan, & Pershing, 2015), and the stressors from an urbanized ocean (e.g., ocean noise, disease, pollution, or repetitive interactions with fishing gear, including the effects of drag from entanglement (van der Hoop et al., 2016; van der Hoop, Corkeron, & Moore, 2017). However, while some or all of these factors may be contributing to reduce calving rates, the causal mechanisms remain unknown.

Other information on the health status of individual right whales informs our understanding of survival and reproduction. As recently reviewed (Kraus et al. 2016), there is a suite of indicators that provide supporting evidence that some anthropogenic threats to North Atlantic right whales are not diminishing and may be getting worse. These indicators include declining overall body condition (Rolland et al., 2016); very high and apparently increasing rates of entanglement in fishing gear (Knowlton et al., 2012); fishing gear that has become heavier and so likely more injurious to whales (Knowlton et al., 2016); and evidence that previous management interventions have not measurably reduced entanglement or entanglement‐related mortality (Pace, Cole, & Henry, 2015). Additionally, recent research has revealed the substantial energy drain on individual whales from drag of ongoing entanglements, which likely results in reduced health and fitness (van der Hoop et al., 2015, 2017). As rates of entanglement in fishing gear appear to be increasing in occurrence and severity (Knowlton et al., 2012, 2016), it is likely that impacts on morbidity are increasing as well. There are also indications that noise from shipping increases the levels of stress hormones in North Atlantic right whales (Rolland et al., 2012), and modeling suggests that their communication space has been reduced substantially by anthropogenic noise (Hatch, Clark, Van Parijs, Frankel, & Ponirakis, 2012).

### 4.3 Data collection, trend detection, and conservation biology of small populations of marine wildlife

A comprehensive time series of photographic identifications of individual animals provides a suite of information beyond determining which animals are alive, where they go, and which females calve each year. In the case of North Atlantic right whales, photo‐identifications have been used to determine individuals' health status (Pettis et al., 2004) and scarring patterns (Knowlton et al., 2012) providing input into the indicators described in the previous section. Other samples, (e.g., skin and blubber biopsy, feces) collected ancillary to photo‐identification sampling from vessels, further inform our understanding of North Atlantic right whales' biology and conservation status (Corkeron, Rolland, Hunt, & Kraus, 2017; Frasier et al., 2007). Through this, photo‐identification‐based monitoring provides a more comprehensive suite of data on a species' status than do other forms of abundance estimation, such as distance sampling‐based surveys (either vessel or aerial, e.g., Hammond et al., 2013).

Thanks to the substantial field efforts made by, and collaborations between, multiple organizations over decades, here we show that we can detect relatively subtle annual changes in the abundance of North Atlantic right whales. Importantly, we demonstrate the capacity to detect multiple inflections in a time series that trended upwards for over two decades but is now flat or possibly declining. Also, we are able to make inference on changes in the abundance of North Atlantic right whales at a time when our capacity to find whales in the field has been reduced, due to both the movement patterns of the whales and the support available to collect field data. In our chosen modeling framework for these data, we can also inform management that, with regard to overall survival, little has changed in 25 years. And for as yet unknown reasons, recruitment (calf production) is not maintaining pace with mortality.

Problems associated with detecting trends in the abundance of marine wildlife populations (Gerrodette, 1987; Taylor, Wade, De Master, & Barlow, 2000) spurred the development of the Potential Biological Removal (PBR) metric as a trigger for management response to anthropogenic mortality of marine mammals (Wade, 1998). The initial work demonstrating the implausibility of detecting a population trend for most small populations of marine mammals (and hence the need to develop the PBR approach) was developed in a null hypothesis significance testing paradigm (Gerrodette, 1987). By relying on a Bayesian approach, we can provide management with a probabilistic statement about the likelihood that the population has declined as opposed to rejecting (or not) a null hypothesis of no decline. In this rare instance, we provide a robust depiction of a species' status. However, the general problem—that uncertainty around abundance estimates will pose problems for detecting trends in small populations—remains. In this case at least, decision makers decide their comfort level with regard to odds of a decline. A further complication is that, while the reduced capacity to identify population trends in other marine mammal populations has been skirted with the PBR approach, the time required to develop and implement mitigation and management actions can take years. For example, the recent designation of Critical Habitat for North Atlantic right whales took over 6 years from when NMFS was first petitioned to act (National Marine Fisheries Service 2015) to designation. Given US legislative requirements, management processes of this sort are inherently slow. Therefore, it is even more important when monitoring very small populations, to be able to detect a change in abundance quickly—whether a decline or an increase—in order to further assess the efficacy of current management actions or develop new ones.

## 5 CONCLUSION

With an estimated abundance of less than 500 individuals, North Atlantic right whales remain one of the most endangered cetaceans (Reilly et al. 2009). Unlike several other baleen whale populations, their population has not been rebounding well in recent decades (Thomas, Reeves, & Brownell, 2016), and our analysis raises concern that the slow recovery has stopped or even reversed. In the two decades since the PBR approach has been in place, enumerated anthropogenic mortalities of North Atlantic right whales have always exceeded PBR (van der Hoop et al., 2013), despite substantial resources directed at addressing this problem (McDonald, Lewison, & Read, 2016; Pace et al., 2015). The purpose of PBR as a limit reference point was to instigate action to mitigate the impact of fishery‐caused mortality on marine mammal populations or species. For North Atlantic right whales, our analysis of their current trend in abundance, coupled with other indicators (Kraus et al. 2016) demonstrates the need for enhanced efforts to address anthropogenic activities causing morbidity and mortality and to maintain the monitoring program that has made this trend analysis possible.

## CONFLICT OF INTEREST

None declared.

## AUTHOR CONTRIBUTIONS

RMP conceived, designed, programmed, interpreted data analysis, and wrote the first draft of the paper. PJC wrote paper. SDK oversaw curation of photo‐identification data; designed, organized, and ran much of the field work contributing to the Catalog and wrote paper.
