# Supplementary material for: State–space mark–recapture estimates reveal a recent decline in abundance of North Atlantic right whales
Source: Ecol Evol. 2017 Sep 18;7(21):8730–41. doi: 10.1002/ece3.3406 (PMC5677501; doi:10.1002/ece3.3406)
Supplement: Supplementary file 1 [file ECE3-7-8730-s001.docx]

Supplementary Material for:

State Space Mark Recapture Estimates Reveal A Recent Decline In Abundance Of North

Atlantic Right Whales

(Pace, Corkeron, and Kraus)

I. Parameter estimate and their characteristics from right whale abundance estimation model

Lower95 Median Upper95 Mean SD MCerr MC%ofSD SSeff AC.10 psrf

phiaf[1] 0.94651 0.96670 0.98065 0.96526 0.00883 0.00015 1.7 3564 0.16826 1.01699

phiaf[2] 0.95582 0.96989 0.98433 0.96975 0.00710 0.00009 1.2 6799 0.07538 1.00465

phiaf[3] 0.93299 0.96274 0.97741 0.95938 0.01245 0.00033 2.6 1458 0.36703 1.00957

phiaf[4] 0.95442 0.96915 0.98332 0.96879 0.00720 0.00007 1 10072 0.07415 1.00285

phiaf[5] 0.95760 0.97050 0.98443 0.97057 0.00675 0.00009 1.4 5203 0.11893 1.00216

phiaf[6] 0.95358 0.96841 0.98191 0.96788 0.00712 0.00006 0.9 12575 0.03634 1.00220

phiaf[7] 0.94820 0.96668 0.97934 0.96547 0.00805 0.00011 1.3 5706 0.10184 1.00356

phiaf[8] 0.95772 0.97059 0.98438 0.97071 0.00664 0.00010 1.5 4206 0.12620 1.00340

phiaf[9] 0.94792 0.96642 0.97920 0.96508 0.00819 0.00011 1.4 5192 0.10803 1.00306

phiaf[10] 0.95168 0.96767 0.98079 0.96688 0.00736 0.00007 1 10975 0.05988 1.00208

phiaf[11] 0.95495 0.96901 0.98193 0.96860 0.00682 0.00006 0.8 14423 0.03352 1.00157

phiaf[12] 0.95263 0.96792 0.97995 0.96723 0.00691 0.00006 0.8 14909 0.02686 1.00289

phiaf[13] 0.95127 0.96718 0.97971 0.96628 0.00725 0.00008 1 9276 0.06364 1.00146

phiaf[14] 0.95522 0.96901 0.98178 0.96868 0.00665 0.00006 0.8 14188 0.04305 1.00244

phiaf[15] 0.95970 0.97168 0.98557 0.97193 0.00656 0.00012 1.9 2904 0.20508 1.00198

phiaf[16] 0.95149 0.96715 0.97916 0.96630 0.00708 0.00008 1.1 8414 0.06425 1.00122

phiaf[17] 0.95337 0.96784 0.97981 0.96719 0.00673 0.00006 0.9 13063 0.03774 1.00228

phiaf[18] 0.96049 0.97183 0.98513 0.97204 0.00628 0.00012 1.9 2654 0.20542 1.00195

phiaf[19] 0.96164 0.97314 0.98671 0.97348 0.00649 0.00016 2.5 1604 0.31662 1.00140

phiaf[20] 0.95912 0.97095 0.98369 0.97105 0.00621 0.00010 1.5 4193 0.14590 1.00245

phiaf[21] 0.95318 0.96788 0.97946 0.96725 0.00667 0.00007 1.1 8665 0.07010 1.00297

phiaf[22] 0.95480 0.96903 0.98188 0.96873 0.00674 0.00009 1.3 5989 0.11600 1.00218

phiaf[23] 0.95331 0.96877 0.98219 0.96828 0.00722 0.00010 1.3 5572 0.15430 1.00328

phiaf[24] 0.95316 0.96897 0.98299 0.96857 0.00737 0.00009 1.3 6071 0.15527 1.00133

phiaf[25] 0.94963 0.96811 0.98322 0.96710 0.00861 0.00010 1.1 8077 0.09951 1.00205

phiam[1] 0.97359 0.98408 0.99074 0.98332 0.00449 0.00007 1.7 3636 0.18520 1.02167

phiam[2] 0.97842 0.98565 0.99279 0.98551 0.00362 0.00004 1.1 7685 0.09095 1.00785

phiam[3] 0.96669 0.98208 0.98951 0.98039 0.00644 0.00016 2.6 1537 0.38466 1.01265

phiam[4] 0.97769 0.98530 0.99205 0.98505 0.00363 0.00004 1 9104 0.08714 1.00469

phiam[5] 0.97926 0.98599 0.99282 0.98591 0.00343 0.00005 1.4 5141 0.14228 1.00373

phiam[6] 0.97711 0.98493 0.99148 0.98460 0.00367 0.00004 1 9598 0.07582 1.00377

phiam[7] 0.97476 0.98409 0.99073 0.98341 0.00418 0.00006 1.5 4539 0.14069 1.00491

phiam[8] 0.97957 0.98605 0.99307 0.98598 0.00339 0.00005 1.4 4930 0.14895 1.00407

phiam[9] 0.97444 0.98394 0.99038 0.98322 0.00424 0.00006 1.5 4306 0.14478 1.00602

phiam[10] 0.97608 0.98458 0.99081 0.98411 0.00380 0.00004 1.1 7686 0.08985 1.00384

phiam[11] 0.97798 0.98524 0.99174 0.98495 0.00350 0.00004 1 9919 0.08201 1.00351

phiam[12] 0.97706 0.98467 0.99092 0.98428 0.00359 0.00004 1 9232 0.07455 1.00548

phiam[13] 0.97607 0.98433 0.99055 0.98381 0.00377 0.00005 1.3 5753 0.11746 1.00335

phiam[14] 0.97818 0.98524 0.99150 0.98500 0.00340 0.00004 1.1 8604 0.06921 1.00408

phiam[15] 0.98032 0.98656 0.99323 0.98658 0.00329 0.00006 1.8 3178 0.21100 1.00332

phiam[16] 0.97619 0.98430 0.99034 0.98383 0.00367 0.00005 1.3 5848 0.12108 1.00322

phiam[17] 0.97714 0.98465 0.99070 0.98427 0.00348 0.00004 1.2 7240 0.09033 1.00426

phiam[18] 0.98058 0.98664 0.99299 0.98663 0.00316 0.00006 1.8 2968 0.21726 1.00326

phiam[19] 0.98129 0.98726 0.99384 0.98733 0.00323 0.00008 2.4 1717 0.32394 1.00178

phiam[20] 0.98021 0.98621 0.99250 0.98615 0.00314 0.00005 1.6 3928 0.16983 1.00281

phiam[21] 0.97730 0.98466 0.99048 0.98431 0.00340 0.00004 1.3 6319 0.11819 1.00452

phiam[22] 0.97786 0.98526 0.99150 0.98502 0.00343 0.00005 1.5 4689 0.16107 1.00219

phiam[23] 0.97722 0.98511 0.99190 0.98479 0.00374 0.00006 1.6 4158 0.20461 1.00518

phiam[24] 0.97735 0.98523 0.99228 0.98494 0.00376 0.00005 1.3 5577 0.17391 1.00230

phiam[25] 0.97470 0.98481 0.99215 0.98418 0.00455 0.00006 1.3 6089 0.14054 1.00421

phi0[1] 0.92093 0.95320 0.97641 0.95102 0.01482 0.00028 1.9 2878 0.31508 1.00442

phi0[2] 0.93350 0.95821 0.98066 0.95729 0.01217 0.00022 1.8 3143 0.25996 1.00012

phi0[3] 0.90238 0.94714 0.97374 0.94291 0.01974 0.00047 2.4 1737 0.42859 1.00253

phi0[4] 0.93085 0.95707 0.97906 0.95593 0.01245 0.00021 1.7 3405 0.27319 1.00061

phi0[5] 0.93522 0.95915 0.98095 0.95838 0.01183 0.00021 1.8 3148 0.29793 1.00043

phi0[6] 0.92914 0.95597 0.97759 0.95462 0.01273 0.00021 1.7 3528 0.26759 1.00040

phi0[7] 0.92309 0.95321 0.97556 0.95131 0.01390 0.00024 1.7 3288 0.27568 1.00117

phi0[8] 0.93573 0.95936 0.98096 0.95861 0.01161 0.00022 1.9 2808 0.29993 1.00067

phi0[9] 0.92206 0.95285 0.97539 0.95073 0.01433 0.00025 1.8 3217 0.29811 1.00037

phi0[10] 0.92698 0.95483 0.97703 0.95322 0.01318 0.00023 1.7 3351 0.27791 1.00056

phi0[11] 0.93129 0.95681 0.97808 0.95563 0.01221 0.00020 1.7 3629 0.27632 1.00067

phi0[12] 0.92821 0.95515 0.97586 0.95374 0.01246 0.00022 1.8 3194 0.27620 1.00033

phi0[13] 0.92666 0.95396 0.97487 0.95246 0.01273 0.00022 1.7 3312 0.27510 1.00046

phi0[14] 0.93223 0.95691 0.97793 0.95576 0.01193 0.00019 1.6 3759 0.27301 1.00072

phi0[15] 0.93848 0.96093 0.98221 0.96035 0.01123 0.00022 1.9 2637 0.33846 1.00066

phi0[16] 0.92744 0.95397 0.97504 0.95250 0.01247 0.00021 1.7 3492 0.28211 1.00071

phi0[17] 0.92981 0.95499 0.97590 0.95373 0.01208 0.00021 1.7 3414 0.27561 1.00061

phi0[18] 0.93903 0.96110 0.98105 0.96050 0.01090 0.00022 2 2466 0.35368 1.00042

phi0[19] 0.94123 0.96299 0.98396 0.96250 0.01101 0.00025 2.3 1890 0.42263 1.00132

phi0[20] 0.93825 0.95987 0.97998 0.95914 0.01085 0.00020 1.9 2852 0.32174 1.00160

phi0[21] 0.92968 0.95512 0.97536 0.95382 0.01194 0.00021 1.8 3202 0.30426 1.00049

phi0[22] 0.93168 0.95696 0.97774 0.95585 0.01195 0.00022 1.8 3083 0.31985 1.00157

phi0[23] 0.93049 0.95655 0.97858 0.95521 0.01267 0.00023 1.8 3010 0.32877 1.00014

phi0[24] 0.93058 0.95681 0.97983 0.95564 0.01266 0.00022 1.8 3208 0.31654 1.00039

phi0[25] 0.92503 0.95547 0.97954 0.95357 0.01441 0.00024 1.6 3734 0.25419 1.00052

phi1[1] 0.93802 0.96227 0.97989 0.96056 0.01102 0.00019 1.7 3314 0.26740 1.00719

phi1[2] 0.94792 0.96611 0.98309 0.96566 0.00891 0.00014 1.6 4010 0.19198 1.00055

phi1[3] 0.92217 0.95747 0.97640 0.95393 0.01515 0.00037 2.4 1675 0.41309 1.00404

phi1[4] 0.94630 0.96524 0.98198 0.96456 0.00909 0.00014 1.5 4464 0.20500 1.00069

phi1[5] 0.94974 0.96687 0.98340 0.96656 0.00862 0.00014 1.6 3771 0.23873 1.00029

phi1[6] 0.94503 0.96438 0.98080 0.96350 0.00927 0.00014 1.6 4146 0.19752 1.00022

phi1[7] 0.94019 0.96224 0.97919 0.96080 0.01023 0.00016 1.6 3917 0.21689 1.00119

phi1[8] 0.94983 0.96702 0.98324 0.96674 0.00845 0.00015 1.7 3348 0.24015 1.00046

phi1[9] 0.93848 0.96194 0.97811 0.96033 0.01054 0.00017 1.6 3826 0.24048 1.00053

phi1[10] 0.94266 0.96350 0.97963 0.96237 0.00961 0.00015 1.5 4293 0.20981 1.00032

phi1[11] 0.94680 0.96502 0.98114 0.96432 0.00884 0.00013 1.5 4655 0.20424 1.00042

phi1[12] 0.94408 0.96376 0.97887 0.96279 0.00901 0.00014 1.5 4224 0.20072 1.00033

phi1[13] 0.94237 0.96284 0.97776 0.96173 0.00925 0.00014 1.5 4301 0.20574 1.00011

phi1[14] 0.94722 0.96509 0.98079 0.96443 0.00860 0.00013 1.5 4689 0.19715 1.00047

phi1[15] 0.95212 0.96828 0.98436 0.96814 0.00819 0.00015 1.9 2854 0.28899 1.00034

phi1[16] 0.94276 0.96283 0.97759 0.96177 0.00901 0.00013 1.5 4616 0.21170 1.00027

phi1[17] 0.94536 0.96366 0.97895 0.96277 0.00869 0.00013 1.5 4572 0.19993 1.00033

phi1[18] 0.95285 0.96848 0.98371 0.96826 0.00790 0.00016 2 2473 0.30414 1.00017

phi1[19] 0.95417 0.96995 0.98567 0.96989 0.00806 0.00019 2.4 1794 0.39291 1.00080

phi1[20] 0.95237 0.96745 0.98272 0.96716 0.00781 0.00014 1.7 3275 0.26089 1.00078

phi1[21] 0.94527 0.96372 0.97830 0.96285 0.00855 0.00014 1.6 3875 0.23358 1.00024

phi1[22] 0.94682 0.96515 0.98073 0.96450 0.00863 0.00015 1.7 3495 0.25839 1.00110

phi1[23] 0.94555 0.96480 0.98127 0.96398 0.00927 0.00016 1.7 3517 0.27994 1.00024

phi1[24] 0.94546 0.96504 0.98214 0.96432 0.00928 0.00015 1.6 3866 0.26237 1.00007

phi1[25] 0.94091 0.96402 0.98229 0.96263 0.01082 0.00016 1.5 4653 0.20166 1.00059

phi2[1] 0.95066 0.96961 0.98287 0.96825 0.00834 0.00014 1.7 3643 0.21905 1.01102

phi2[2] 0.95922 0.97258 0.98586 0.97238 0.00667 0.00009 1.3 5526 0.12241 1.00166

phi2[3] 0.93794 0.96585 0.97994 0.96283 0.01179 0.00029 2.5 1616 0.39845 1.00612

phi2[4] 0.95819 0.97187 0.98510 0.97149 0.00677 0.00008 1.3 6337 0.13263 1.00118

phi2[5] 0.96099 0.97317 0.98632 0.97311 0.00640 0.00009 1.4 4766 0.17626 1.00055

phi2[6] 0.95693 0.97125 0.98399 0.97064 0.00688 0.00008 1.2 6585 0.12249 1.00042

phi2[7] 0.95207 0.96962 0.98181 0.96844 0.00768 0.00011 1.5 4609 0.15840 1.00157

phi2[8] 0.96060 0.97328 0.98572 0.97325 0.00628 0.00010 1.6 3928 0.17752 1.00065

phi2[9] 0.95149 0.96937 0.98153 0.96806 0.00790 0.00012 1.5 4457 0.18078 1.00122

phi2[10] 0.95488 0.97055 0.98284 0.96972 0.00713 0.00009 1.3 6157 0.13689 1.00044

phi2[11] 0.95835 0.97176 0.98399 0.97130 0.00653 0.00008 1.2 7179 0.12596 1.00052

phi2[12] 0.95626 0.97077 0.98221 0.97005 0.00665 0.00008 1.2 6455 0.11811 1.00084

phi2[13] 0.95460 0.97007 0.98125 0.96919 0.00686 0.00009 1.3 5806 0.13360 1.00013

phi2[14] 0.95878 0.97179 0.98382 0.97139 0.00633 0.00008 1.2 6995 0.11365 1.00061

phi2[15] 0.96278 0.97425 0.98685 0.97439 0.00609 0.00011 1.9 2814 0.23711 1.00039

phi2[16] 0.95569 0.97007 0.98169 0.96922 0.00666 0.00008 1.2 6782 0.13709 1.00017

phi2[17] 0.95669 0.97068 0.98158 0.97004 0.00639 0.00008 1.2 6572 0.11762 1.00048

phi2[18] 0.96315 0.97442 0.98609 0.97449 0.00585 0.00011 2 2598 0.25021 1.00028

phi2[19] 0.96461 0.97561 0.98805 0.97580 0.00602 0.00015 2.4 1705 0.35989 1.00047

phi2[20] 0.96257 0.97362 0.98521 0.97359 0.00575 0.00009 1.6 3933 0.19480 1.00057

phi2[21] 0.95716 0.97073 0.98163 0.97010 0.00625 0.00008 1.3 5491 0.15394 1.00046

phi2[22] 0.95856 0.97183 0.98395 0.97144 0.00636 0.00010 1.5 4341 0.19164 1.00080

phi2[23] 0.95740 0.97155 0.98457 0.97101 0.00692 0.00011 1.6 3992 0.22912 1.00084

phi2[24] 0.95654 0.97175 0.98457 0.97130 0.00694 0.00010 1.4 4898 0.20614 1.00003

phi2[25] 0.95319 0.97096 0.98529 0.96991 0.00828 0.00011 1.3 5839 0.15329 1.00105

phi3[1] 0.96057 0.97553 0.98580 0.97441 0.00651 0.00010 1.6 3896 0.18232 1.01544

phi3[2] 0.96776 0.97784 0.98857 0.97776 0.00518 0.00006 1.2 7120 0.07169 1.00352

phi3[3] 0.94991 0.97259 0.98315 0.97000 0.00938 0.00024 2.5 1561 0.38747 1.00855

phi3[4] 0.96676 0.97731 0.98773 0.97705 0.00523 0.00005 1 9779 0.07657 1.00216

phi3[5] 0.96913 0.97834 0.98881 0.97836 0.00494 0.00007 1.3 5562 0.12869 1.00133

phi3[6] 0.96554 0.97679 0.98663 0.97636 0.00530 0.00005 1 10066 0.06382 1.00117

phi3[7] 0.96172 0.97555 0.98496 0.97456 0.00598 0.00008 1.4 5469 0.11770 1.00243

phi3[8] 0.96911 0.97840 0.98865 0.97847 0.00485 0.00007 1.5 4700 0.13093 1.00142

phi3[9] 0.96124 0.97535 0.98470 0.97427 0.00613 0.00009 1.4 4955 0.13544 1.00258

phi3[10] 0.96381 0.97627 0.98553 0.97561 0.00549 0.00006 1.1 8924 0.07963 1.00116

phi3[11] 0.96682 0.97722 0.98673 0.97689 0.00501 0.00005 0.9 11467 0.06479 1.00115

phi3[12] 0.96527 0.97642 0.98546 0.97588 0.00511 0.00005 0.9 11106 0.05372 1.00207

phi3[13] 0.96384 0.97591 0.98459 0.97518 0.00532 0.00006 1.1 8022 0.08272 1.00077

phi3[14] 0.96705 0.97723 0.98641 0.97696 0.00485 0.00004 0.9 12066 0.04803 1.00138

phi3[15] 0.97045 0.97917 0.98910 0.97939 0.00472 0.00009 1.9 2843 0.19822 1.00100

phi3[16] 0.96448 0.97589 0.98462 0.97520 0.00514 0.00006 1.1 8711 0.08388 1.00071

phi3[17] 0.96536 0.97637 0.98466 0.97586 0.00491 0.00005 1 10088 0.05603 1.00133

phi3[18] 0.97098 0.97931 0.98868 0.97947 0.00451 0.00008 1.9 2835 0.20801 1.00093

phi3[19] 0.97188 0.98026 0.98988 0.98053 0.00467 0.00011 2.4 1692 0.33184 1.00053

phi3[20] 0.97036 0.97866 0.98781 0.97874 0.00443 0.00007 1.6 3911 0.14527 1.00086

phi3[21] 0.96601 0.97640 0.98483 0.97592 0.00478 0.00006 1.2 7267 0.09137 1.00141

phi3[22] 0.96709 0.97726 0.98675 0.97700 0.00489 0.00007 1.4 5319 0.14055 1.00086

phi3[23] 0.96581 0.97704 0.98714 0.97665 0.00536 0.00008 1.5 4546 0.19133 1.00203

phi3[24] 0.96581 0.97720 0.98771 0.97688 0.00539 0.00007 1.3 5922 0.16383 1.00042

phi3[25] 0.96241 0.97661 0.98796 0.97575 0.00653 0.00008 1.2 6873 0.12184 1.00193

phi4[1] 0.96828 0.98028 0.98876 0.97936 0.00529 0.00008 1.6 3967 0.16993 1.01934

phi4[2] 0.97384 0.98215 0.99082 0.98206 0.00422 0.00005 1.1 8651 0.06041 1.00578

phi4[3] 0.95944 0.97789 0.98649 0.97576 0.00767 0.00020 2.6 1520 0.38261 1.01088

phi4[4] 0.97318 0.98171 0.99025 0.98149 0.00425 0.00004 0.9 11156 0.05975 1.00345

phi4[5] 0.97482 0.98256 0.99084 0.98255 0.00401 0.00005 1.3 5687 0.11609 1.00250

phi4[6] 0.97227 0.98129 0.98938 0.98093 0.00429 0.00004 0.9 12678 0.04630 1.00240

phi4[7] 0.96894 0.98029 0.98784 0.97948 0.00488 0.00007 1.4 5081 0.11145 1.00365

phi4[8] 0.97519 0.98261 0.99106 0.98264 0.00395 0.00006 1.4 5130 0.12051 1.00268

phi4[9] 0.96877 0.98012 0.98773 0.97924 0.00498 0.00007 1.4 4777 0.12212 1.00435

phi4[10] 0.97099 0.98087 0.98848 0.98033 0.00445 0.00005 1 9655 0.06193 1.00242

phi4[11] 0.97326 0.98166 0.98947 0.98137 0.00407 0.00004 0.9 13402 0.04831 1.00227

phi4[12] 0.97199 0.98098 0.98833 0.98055 0.00416 0.00004 0.9 12783 0.03752 1.00381

phi4[13] 0.97078 0.98057 0.98773 0.97997 0.00436 0.00005 1.1 8347 0.07734 1.00198

phi4[14] 0.97342 0.98166 0.98908 0.98143 0.00394 0.00003 0.9 13531 0.03126 1.00269

phi4[15] 0.97594 0.98322 0.99112 0.98339 0.00384 0.00007 1.8 2948 0.18872 1.00208

phi4[16] 0.97117 0.98055 0.98761 0.97999 0.00422 0.00005 1.1 7833 0.07868 1.00187

phi4[17] 0.97213 0.98095 0.98788 0.98053 0.00401 0.00004 1 10819 0.04622 1.00277

phi4[18] 0.97670 0.98333 0.99115 0.98345 0.00367 0.00007 1.9 2871 0.19594 1.00203

phi4[19] 0.97735 0.98409 0.99205 0.98431 0.00379 0.00009 2.5 1648 0.31867 1.00101

phi4[20] 0.97608 0.98281 0.99039 0.98286 0.00362 0.00006 1.6 4045 0.13644 1.00171

phi4[21] 0.97256 0.98097 0.98784 0.98058 0.00390 0.00004 1.1 7571 0.07810 1.00296

phi4[22] 0.97333 0.98167 0.98929 0.98146 0.00398 0.00005 1.3 5593 0.12923 1.00138

phi4[23] 0.97216 0.98150 0.98959 0.98117 0.00437 0.00006 1.5 4670 0.18240 1.00363

phi4[24] 0.97261 0.98164 0.99034 0.98136 0.00439 0.00006 1.3 6216 0.15215 1.00125

phi4[25] 0.96962 0.98115 0.99045 0.98043 0.00535 0.00006 1.2 7093 0.11758 1.00308

BetaAge 0.11608 0.22145 0.32395 0.22016 0.05327 0.00122 2.3 1903 0.53256 1.00508

BetaSex[1] 2.67494 3.09106 3.52807 3.09819 0.21812 0.00490 2.2 1984 0.50110 1.00058

BetaSex[2] -1.12258-0.75558-0.40269 -0.75555 0.18331 0.00264 1.4 4829 0.16648 1.00153

pcap1[1] 0.55525 0.62700 0.69257 0.62670 0.03498 0.00078 2.2 1998 0.51746 1.00351

pcap1[2] 0.39091 0.47838 0.56422 0.47864 0.04433 0.00053 1.2 7019 0.12933 1.00232

pcap1[3] 0.61066 0.69131 0.76413 0.68997 0.03941 0.00047 1.2 7081 0.12999 1.00126

pcap1[4] 0.74333 0.80512 0.85991 0.80360 0.03006 0.00036 1.2 6880 0.12858 1.00115

pcap1[5] 0.78486 0.83896 0.88767 0.83736 0.02649 0.00030 1.1 7825 0.12237 1.00129

pcap1[6] 0.72279 0.78550 0.84323 0.78410 0.03101 0.00037 1.2 6962 0.12513 1.00098

pcap1[7] 0.80721 0.85652 0.90057 0.85510 0.02409 0.00027 1.1 7962 0.11435 1.00081

pcap1[8] 0.70730 0.77325 0.83391 0.77176 0.03251 0.00038 1.2 7507 0.12733 1.00069

pcap1[9] 0.75280 0.81123 0.86534 0.80968 0.02900 0.00034 1.2 7339 0.13171 1.00116

pcap1[10] 0.76975 0.82534 0.87600 0.82387 0.02738 0.00032 1.2 7550 0.12533 1.00075

pcap1[11] 0.87660 0.91099 0.94220 0.90981 0.01695 0.00019 1.1 8369 0.11123 1.00083

pcap1[12] 0.88133 0.91489 0.94333 0.91372 0.01603 0.00018 1.1 8000 0.11635 1.00065

pcap1[13] 0.89231 0.92279 0.94959 0.92171 0.01487 0.00016 1.1 8509 0.09862 1.00047

pcap1[14] 0.76965 0.82362 0.87047 0.82224 0.02593 0.00031 1.2 7038 0.13622 1.00070

pcap1[15] 0.91479 0.93997 0.96165 0.93903 0.01221 0.00013 1 9360 0.09774 1.00059

pcap1[16] 0.87717 0.91037 0.93950 0.90926 0.01608 0.00018 1.1 8099 0.11567 1.00056

pcap1[17] 0.92405 0.94700 0.96687 0.94603 0.01115 0.00012 1 9198 0.11012 1.00044

pcap1[18] 0.89326 0.92296 0.94815 0.92193 0.01419 0.00016 1.1 7835 0.11818 1.00063

pcap1[19] 0.92152 0.94466 0.96423 0.94382 0.01107 0.00013 1.2 7489 0.11110 1.00061

pcap1[20] 0.88457 0.91514 0.94169 0.91419 0.01478 0.00018 1.2 7091 0.12693 1.00048

pcap1[21] 0.91963 0.94318 0.96292 0.94229 0.01123 0.00013 1.1 7718 0.12451 1.00023

pcap1[22] 0.76325 0.81502 0.86117 0.81380 0.02516 0.00033 1.3 5770 0.16283 1.00040

pcap1[23] 0.54127 0.61402 0.68222 0.61345 0.03622 0.00049 1.3 5495 0.17815 1.00042

pcap1[24] 0.76047 0.81439 0.86275 0.81316 0.02634 0.00037 1.4 5173 0.19836 1.00021

pcap1[25] 0.44683 0.52210 0.59946 0.52216 0.03906 0.00055 1.4 5098 0.18079 1.00011

pcap2[1] 0.61319 0.67810 0.73851 0.67745 0.03196 0.00104 3.3 936 0.72968 1.00237

pcap2[2] 0.45060 0.53471 0.61852 0.53439 0.04303 0.00059 1.4 5234 0.16378 1.00129

pcap2[3] 0.66812 0.73698 0.80369 0.73573 0.03470 0.00049 1.4 4932 0.16088 1.00062

pcap2[4] 0.78691 0.83792 0.88431 0.83666 0.02512 0.00033 1.3 5923 0.14681 1.00059

pcap2[5] 0.82192 0.86702 0.90630 0.86571 0.02180 0.00027 1.3 6369 0.14449 1.00069

pcap2[6] 0.76705 0.82106 0.86968 0.81965 0.02648 0.00037 1.4 5204 0.16179 1.00034

pcap2[7] 0.84133 0.88200 0.91803 0.88080 0.01980 0.00025 1.2 6404 0.14055 1.00025

pcap2[8] 0.75407 0.81017 0.86060 0.80888 0.02746 0.00036 1.3 5807 0.15833 1.00022

pcap2[9] 0.79394 0.84314 0.88794 0.84191 0.02421 0.00032 1.3 5880 0.15982 1.00050

pcap2[10] 0.80819 0.85551 0.89670 0.85415 0.02277 0.00030 1.3 5599 0.15655 1.00022

pcap2[11] 0.89926 0.92764 0.95193 0.92667 0.01363 0.00016 1.2 6908 0.12886 1.00034

pcap2[12] 0.90446 0.93087 0.95424 0.92991 0.01283 0.00015 1.2 7070 0.13730 1.00021

pcap2[13] 0.91252 0.93738 0.95868 0.93649 0.01198 0.00014 1.2 7090 0.12634 1.00021

pcap2[14] 0.80886 0.85381 0.89282 0.85275 0.02152 0.00030 1.4 5256 0.16968 1.00020

pcap2[15] 0.93150 0.95148 0.96897 0.95072 0.00974 0.00011 1.1 7623 0.11975 1.00017

pcap2[16] 0.90022 0.92706 0.95021 0.92621 0.01295 0.00016 1.3 6301 0.14290 1.00012

pcap2[17] 0.93880 0.95715 0.97258 0.95647 0.00876 0.00010 1.1 8033 0.11635 1.00008

pcap2[18] 0.91402 0.93749 0.95753 0.93670 0.01125 0.00013 1.2 7155 0.13378 1.00020

pcap2[19] 0.93708 0.95528 0.97080 0.95465 0.00875 0.00010 1.1 7709 0.11542 1.00027

pcap2[20] 0.90650 0.93114 0.95187 0.93032 0.01171 0.00015 1.3 5964 0.14881 1.00014

pcap2[21] 0.93567 0.95411 0.97020 0.95339 0.00896 0.00012 1.3 5854 0.14526 1.00006

pcap2[22] 0.80397 0.84653 0.88463 0.84551 0.02076 0.00030 1.5 4727 0.19828 1.00013

pcap2[23] 0.60033 0.66578 0.72623 0.66507 0.03244 0.00052 1.6 3944 0.22791 1.00007

pcap2[24] 0.80200 0.84593 0.88690 0.84494 0.02187 0.00034 1.6 4031 0.24215 1.00024

pcap2[25] 0.50668 0.57788 0.64919 0.57749 0.03647 0.00058 1.6 3981 0.22136 1.00010

pie 0.42806 0.46659 0.50672 0.46667 0.02003 0.00011 0.6 32010 0.01292 1.00029

AlphaSex[1] 0.45536 0.74505 1.03271 0.74592 0.14698 0.00481 3.3 934 0.72992 1.00256

AlphaSex[2] -0.53136-0.22443 0.06392 -0.22498 0.15153 0.00367 2.4 1700 0.56339 1.00029

AlphaTime[1] 0.00000 0.00000 0.00000 0.00000 0.00000 NA NA NA NA NA

AlphaTime[2] 0.00000 0.00000 0.00000 0.00000 0.00000 NA NA NA NA NA

AlphaTime[3] -0.95452-0.60593-0.26027 -0.60713 0.17721 0.00279 1.6 4022 0.20108 1.00002

AlphaTime[4] -0.070330.28534 0.64869 0.28551 0.18383 0.00295 1.6 3883 0.20561 1.00015

AlphaTime[5] 0.52717 0.89931 1.26610 0.89906 0.18862 0.00286 1.5 4349 0.19977 1.00017

AlphaTime[6] 0.74353 1.12955 1.50073 1.13054 0.19318 0.00282 1.5 4701 0.19228 1.00010

AlphaTime[7] 0.42223 0.77846 1.14631 0.77835 0.18490 0.00285 1.5 4206 0.21130 1.00034

AlphaTime[8] 0.88881 1.26610 1.64979 1.26765 0.19454 0.00296 1.5 4309 0.19699 1.00035

AlphaTime[9] 0.34503 0.70603 1.07402 0.70658 0.18636 0.00303 1.6 3794 0.22682 1.00049

AlphaTime[10] 0.57132 0.93708 1.30749 0.93796 0.18807 0.00290 1.5 4216 0.21005 1.00020

AlphaTime[11] 0.66849 1.03317 1.41015 1.03342 0.18950 0.00301 1.6 3968 0.21489 1.00036

AlphaTime[12] 1.41120 1.80580 2.22022 1.80782 0.20690 0.00295 1.4 4904 0.17685 1.00022

AlphaTime[13] 1.45484 1.85432 2.25729 1.85600 0.20469 0.00309 1.5 4385 0.19535 1.00030

AlphaTime[14] 1.54814 1.96054 2.36315 1.96271 0.20790 0.00311 1.5 4473 0.18055 1.00056

AlphaTime[15] 0.66450 1.02029 1.37861 1.02083 0.18194 0.00307 1.7 3522 0.25327 1.00052

AlphaTime[16] 1.80886 2.23009 2.65501 2.23330 0.21612 0.00307 1.4 4945 0.17453 1.00041

AlphaTime[17] 1.42887 1.79810 2.20435 1.79918 0.19788 0.00302 1.5 4294 0.21215 1.00048

AlphaTime[18] 1.94173 2.36338 2.80404 2.36396 0.21972 0.00318 1.4 4781 0.18443 1.00042

AlphaTime[19] 1.56236 1.96364 2.34739 1.96418 0.20043 0.00320 1.6 3927 0.21681 1.00053

AlphaTime[20] 1.91122 2.31773 2.74860 2.31963 0.21326 0.00332 1.6 4116 0.19671 1.00064

AlphaTime[21] 1.47824 1.85820 2.23762 1.85968 0.19340 0.00337 1.7 3288 0.24043 1.00102

AlphaTime[22] 1.87006 2.28909 2.70267 2.29065 0.21210 0.00346 1.6 3754 0.21917 1.00106

AlphaTime[23] 0.62623 0.96229 1.30810 0.96266 0.17370 0.00335 1.9 2689 0.30952 1.00144

AlphaTime[24] -0.37560-0.05610 0.26699 -0.05645 0.16437 0.00345 2.1 2266 0.35824 1.00143

AlphaTime[25] 0.60333 0.95775 1.32156 0.95920 0.18277 0.00368 2 2464 0.34335 1.00235

AlphaTime[26] -0.77269-0.43067-0.11031 -0.43170 0.16825 0.00352 2.1 2291 0.35199 1.00199

sigma 0.00117 0.19423 0.44505 0.20939 0.12997 0.00518 4 630 0.79342 1.00359

epsilon 1.27570 1.40537 1.54236 1.40753 0.06824 0.00110 1.6 3866 0.27651 1.00294

gamma[1] 0.28413 0.31547 0.34734 0.31558 0.01617 0.00015 0.9 12034 0.03407 1.00824

gamma[2] 0.01010 0.02038 0.03308 0.02094 0.00602 0.00003 0.6 30249 0.00067 1.00078

gamma[3] 0.01452 0.02610 0.04025 0.02663 0.00672 0.00004 0.6 29910 -0.000571.00119

gamma[4] 0.01277 0.02471 0.03828 0.02522 0.00663 0.00004 0.5 34157 0.00652 1.00035

gamma[5] 0.01301 0.02515 0.03885 0.02570 0.00673 0.00004 0.5 33969 0.01296 1.00012

gamma[6] 0.00819 0.01823 0.03051 0.01881 0.00587 0.00003 0.6 31702 -0.001031.00014

gamma[7] 0.02759 0.04359 0.06257 0.04417 0.00904 0.00005 0.6 32456 -0.007461.00036

gamma[8] 0.02027 0.03539 0.05240 0.03603 0.00837 0.00005 0.6 31131 0.00445 1.00050

gamma[9] 0.00236 0.00975 0.01939 0.01041 0.00461 0.00003 0.6 26540 0.00722 1.00012

gamma[10] 0.00643 0.01601 0.02857 0.01669 0.00585 0.00004 0.6 27649 0.00834 1.00022

gamma[11] 0.01200 0.02474 0.03948 0.02539 0.00720 0.00004 0.6 32183 0.00338 1.00004

gamma[12] 0.03521 0.05544 0.07730 0.05612 0.01080 0.00006 0.5 34286 -0.000731.00083

gamma[13] 0.05070 0.07448 0.09975 0.07514 0.01261 0.00007 0.5 33676 -0.001831.00057

gamma[14] 0.02715 0.04596 0.06783 0.04674 0.01053 0.00006 0.5 34712 0.00702 1.00064

gamma[15] 0.03285 0.05337 0.07756 0.05415 0.01159 0.00006 0.6 32609 0.00039 1.00044

gamma[16] 0.04711 0.07231 0.09976 0.07307 0.01358 0.00007 0.5 34300 0.00041 1.00029

gamma[17] 0.03652 0.06038 0.08711 0.06136 0.01311 0.00007 0.5 33272 0.00142 1.00063

gamma[18] 0.05154 0.07952 0.10999 0.08041 0.01509 0.00008 0.5 34376 0.00226 1.00099

gamma[19] 0.07039 0.10325 0.13910 0.10419 0.01765 0.00009 0.5 35518 0.00699 1.00179

gamma[20] 0.06893 0.10397 0.14123 0.10494 0.01871 0.00010 0.5 33716 0.01411 1.00075

gamma[21] 0.06379 0.09961 0.13945 0.10078 0.01962 0.00011 0.6 30832 0.00929 1.00156

gamma[22] 0.02656 0.05487 0.08713 0.05624 0.01582 0.00009 0.6 29142 0.01072 1.00120

gamma[23] 0.01902 0.04515 0.07734 0.04672 0.01537 0.00010 0.7 23079 0.01340 1.00066

gamma[24] 0.01760 0.04396 0.07709 0.04566 0.01573 0.00013 0.8 15752 0.03332 1.00040

gamma[25] 0.00231 0.01685 0.04006 0.01882 0.01067 0.00008 0.8 16545 0.01888 1.00016

gamma[26] 0.00000 0.00694 0.02964 0.00997 0.00998 0.00012 1.2 7015 0.11011 1.00013

b[1] 0.35842 0.39490 0.43175 0.39503 0.01881 0.00012 0.6 24991 0.02372 1.00246

b[2] 0.00856 0.01745 0.02830 0.01794 0.00517 0.00003 0.6 30356 0.00049 1.00020

b[3] 0.01203 0.02187 0.03369 0.02234 0.00565 0.00003 0.6 29985 -0.002511.00029

b[4] 0.01048 0.02015 0.03130 0.02059 0.00542 0.00003 0.5 34244 0.00370 1.00001

b[5] 0.01054 0.02001 0.03115 0.02046 0.00538 0.00003 0.5 34077 0.01257 1.00008

b[6] 0.00627 0.01414 0.02363 0.01459 0.00456 0.00003 0.6 32007 -0.002691.00001

b[7] 0.02082 0.03315 0.04759 0.03361 0.00692 0.00004 0.6 32593 -0.007141.00009

b[8] 0.01486 0.02575 0.03838 0.02620 0.00611 0.00003 0.6 32009 0.00410 1.00006

b[9] 0.00179 0.00683 0.01376 0.00730 0.00324 0.00002 0.6 26705 0.00692 1.00020

b[10] 0.00427 0.01112 0.01965 0.01158 0.00407 0.00002 0.6 27866 0.00898 0.99999

b[11] 0.00828 0.01687 0.02703 0.01732 0.00493 0.00003 0.6 32315 0.00058 1.00021

b[12] 0.02374 0.03686 0.05192 0.03731 0.00724 0.00004 0.5 34625 -0.004531.00000

b[13] 0.03204 0.04672 0.06317 0.04715 0.00803 0.00004 0.5 34002 -0.000301.00035

b[14] 0.01578 0.02665 0.03949 0.02712 0.00616 0.00003 0.5 34910 0.00572 1.00002

b[15] 0.01759 0.02948 0.04259 0.02995 0.00647 0.00004 0.5 33227 -0.000111.00012

b[16] 0.02448 0.03782 0.05245 0.03823 0.00724 0.00004 0.5 34434 0.00169 1.00031

b[17] 0.01775 0.02929 0.04266 0.02976 0.00647 0.00004 0.5 33589 -0.000071.00012

b[18] 0.02330 0.03616 0.05058 0.03661 0.00704 0.00004 0.5 34628 -0.000741.00011

b[19] 0.02938 0.04317 0.05899 0.04362 0.00762 0.00004 0.5 36426 0.00383 1.00040

b[20] 0.02550 0.03892 0.05384 0.03936 0.00731 0.00004 0.5 34410 0.00440 1.00048

b[21] 0.02067 0.03342 0.04706 0.03383 0.00682 0.00004 0.6 32928 0.00093 1.00006

b[22] 0.00811 0.01651 0.02672 0.01697 0.00487 0.00003 0.6 31793 0.00380 1.00003

b[23] 0.00522 0.01283 0.02205 0.01330 0.00444 0.00003 0.6 26501 0.00731 1.00014

b[24] 0.00476 0.01190 0.02119 0.01240 0.00434 0.00003 0.7 19109 0.02655 1.00010

b[25] 0.00055 0.00436 0.01036 0.00488 0.00278 0.00002 0.8 17645 0.01500 1.00008

b[26] 0.00000 0.00176 0.00755 0.00253 0.00254 0.00003 1.2 7146 0.10502 1.00013

N[1] 265.0 270.0 276.0 270.4 2.95578 0.31993 10.8 85 0.87258 1.27320

N[2] 268.0 272.0 278.0 272.6 2.80593 0.10630 3.8 697 0.38328 1.10422

N[3] 276.0 282.0 286.0 281.8 2.59283 0.07677 3 1141 0.32770 1.07513

N[4] 277.0 281.0 286.0 281.5 2.39094 0.04518 1.9 2800 0.23091 1.02299

N[5] 285.0 288.0 292.0 288.3 1.83329 0.03436 1.9 2846 0.25894 1.01804

N[6] 290.0 293.0 296.0 292.8 1.62627 0.03042 1.9 2857 0.26303 1.01345

N[7] 305.0 308.0 311.0 307.9 1.83114 0.03264 1.8 3146 0.23585 1.00789

N[8] 312.0 315.0 318.0 315.3 1.77486 0.02980 1.7 3548 0.21871 1.00422

N[9] 311.0 314.0 317.0 314.1 1.69300 0.02610 1.5 4209 0.20814 1.00193

N[10] 308.0 311.0 314.0 311.1 1.69709 0.02465 1.5 4739 0.15128 1.00128

N[11] 312.0 314.0 316.0 313.8 1.34168 0.02442 1.8 3018 0.20188 1.00145

N[12] 331.0 332.0 334.0 332.3 1.12346 0.02540 2.3 1957 0.25721 1.00299

N[13] 354.0 355.0 358.0 355.4 1.19040 0.02622 2.2 2061 0.24769 1.00252

N[14] 361.0 363.0 365.0 363.0 1.34899 0.02532 1.9 2838 0.16033 1.00152

N[15] 372.0 375.0 377.0 374.8 1.50942 0.02732 1.8 3053 0.17183 1.00117

N[16] 394.0 396.0 398.0 395.8 1.26802 0.03077 2.4 1698 0.26079 1.00137

N[17] 402.0 404.0 407.0 404.5 1.42826 0.03518 2.5 1648 0.24172 1.00162

N[18] 417.0 419.0 421.0 418.9 1.35012 0.04505 3.3 898 0.37537 1.00173

N[19] 441.0 443.0 446.0 443.4 1.43497 0.05047 3.5 808 0.43558 1.00429

N[20] 464.0 466.0 469.0 466.2 1.45171 0.05424 3.7 716 0.52899 1.00652

N[21] 479.0 482.0 486.0 482.5 1.99050 0.07192 3.6 766 0.52164 1.00485

N[22] 477.0 481.0 485.0 481.1 2.29558 0.08217 3.6 780 0.62241 1.00242

N[23] 472.0 479.0 485.0 479.0 3.40122 0.10397 3.1 1070 0.59792 1.00266

N[24] 468.0 476.0 485.0 476.2 4.59532 0.13586 3 1144 0.61585 1.00477

N[25] 457.0 468.0 478.0 468.4 5.52968 0.15451 2.8 1281 0.62076 1.00447

N[26] 444.0 458.0 471.0 458.0 7.06746 0.16125 2.3 1921 0.44248 1.00509

NM[1] 143.0 147.0 152.0 147.4 2.56166 0.04663 1.8 3019 0.12084 1.03055

NM[2] 144.0 149.0 153.0 149.0 2.47526 0.02729 1.1 8226 0.07366 1.01232

NM[3] 151.0 155.0 160.0 155.3 2.47036 0.02498 1 9778 0.06849 1.00890

NM[4] 154.0 159.0 163.0 158.6 2.45713 0.02076 0.8 14006 0.04796 1.00226

NM[5] 156.0 161.0 165.0 161.0 2.35012 0.01981 0.8 14069 0.05080 1.00120

NM[6] 160.0 164.0 168.0 164.4 2.25074 0.01792 0.8 15780 0.04959 1.00096

NM[7] 164.0 169.0 173.0 169.4 2.35827 0.01837 0.8 16473 0.04980 1.00082

NM[8] 172.0 177.0 181.0 176.6 2.34219 0.01727 0.7 18398 0.03994 1.00052

NM[9] 174.0 179.0 183.0 178.6 2.33787 0.01695 0.7 19032 0.03589 1.00033

NM[10] 173.0 179.0 182.0 178.5 2.33691 0.01629 0.7 20573 0.02969 1.00047

NM[11] 178.0 183.0 186.0 182.7 2.18204 0.01646 0.8 17570 0.02937 1.00026

NM[12] 188.0 192.0 196.0 191.7 2.29112 0.01783 0.8 16504 0.03895 1.00035

NM[13] 199.0 204.0 208.0 204.3 2.44442 0.01906 0.8 16442 0.03412 1.00017

NM[14] 205.0 210.0 214.0 210.0 2.50538 0.01791 0.7 19571 0.02701 1.00014

NM[15] 213.0 218.0 222.0 217.9 2.50269 0.01836 0.7 18578 0.03155 1.00020

NM[16] 226.0 231.0 236.0 230.9 2.72033 0.02005 0.7 18413 0.03492 1.00004

NM[17] 235.0 240.0 245.0 240.4 2.77116 0.02082 0.8 17713 0.03172 1.00009

NM[18] 248.0 254.0 258.0 253.6 2.69053 0.02080 0.8 16729 0.03779 1.00010

NM[19] 260.0 265.0 270.0 264.9 2.79970 0.02475 0.9 12791 0.04852 1.00090

NM[20] 269.0 274.0 279.0 274.0 2.72847 0.02810 1 9425 0.06140 1.00131

NM[21] 277.0 283.0 288.0 282.9 2.99500 0.04527 1.5 4377 0.10626 1.00190

NM[22] 275.0 281.0 287.0 281.3 3.16768 0.06514 2.1 2365 0.17807 1.00207

NM[23] 275.0 281.0 288.0 281.2 3.52354 0.07795 2.2 2043 0.26779 1.00339

NM[24] 271.0 279.0 287.0 279.3 4.18812 0.09835 2.3 1813 0.36018 1.00619

NM[25] 268.0 277.0 285.0 276.9 4.53169 0.10606 2.3 1826 0.40165 1.00651

NM[26] 261.0 272.0 282.0 272.3 5.43265 0.11605 2.1 2191 0.33875 1.00695

NF[1] 116.0 123.0 128.0 123.0 3.12732 0.13524 4.3 535 0.33334 1.08988

NF[2] 118.0 124.0 129.0 123.7 2.97412 0.06409 2.2 2154 0.17692 1.03824

NF[3] 120.0 126.0 131.0 126.4 2.86532 0.05125 1.8 3125 0.14974 1.02422

NF[4] 118.0 123.0 128.0 122.9 2.82200 0.03667 1.3 5923 0.11537 1.00707

NF[5] 123.0 127.0 132.0 127.3 2.52525 0.02992 1.2 7122 0.10105 1.00410

NF[6] 124.0 128.0 133.0 128.4 2.44764 0.02775 1.1 7782 0.08505 1.00216

NF[7] 134.0 138.0 143.0 138.5 2.54626 0.02803 1.1 8253 0.08738 1.00126

NF[8] 133.0 139.0 143.0 138.7 2.69297 0.02639 1 10416 0.06838 1.00047

NF[9] 130.0 135.0 140.0 135.4 2.62605 0.02464 0.9 11358 0.06823 1.00013

NF[10] 128.0 133.0 137.0 132.6 2.49094 0.02303 0.9 11699 0.05871 1.00006

NF[11] 127.0 131.0 135.0 131.2 2.30019 0.02036 0.9 12764 0.05604 1.00008

NF[12] 136.0 141.0 145.0 140.6 2.35515 0.02211 0.9 11346 0.05898 1.00013

NF[13] 147.0 151.0 156.0 151.1 2.51973 0.02198 0.9 13141 0.05004 1.00021

NF[14] 147.0 153.0 157.0 153.0 2.62428 0.02110 0.8 15476 0.03960 1.00009

NF[15] 152.0 157.0 162.0 156.9 2.70731 0.02489 0.9 11832 0.05027 1.00013

NF[16] 159.0 165.0 170.0 165.0 2.84124 0.02971 1 9146 0.06017 1.00016

NF[17] 158.0 164.0 169.0 164.0 2.95335 0.03313 1.1 7945 0.06521 1.00021

NF[18] 159.0 165.0 170.0 165.3 2.90200 0.04019 1.4 5213 0.08864 1.00017

N[19] 173.0 178.0 184.0 178.5 2.95340 0.04212 1.4 4916 0.09573 1.00017

NF[20] 186.0 192.0 197.0 192.2 2.86382 0.04534 1.6 3990 0.11464 1.00023

NF[21] 193.0 200.0 205.0 199.6 3.10130 0.05409 1.7 3288 0.14866 1.00044

NF[22] 194.0 200.0 206.0 199.7 3.25620 0.05996 1.8 2949 0.18382 1.00011

NF[23] 190.0 198.0 204.0 197.7 3.68274 0.07177 1.9 2633 0.26303 1.00067

NF[24] 188.0 197.0 204.0 196.9 4.16279 0.08146 2 2612 0.31270 1.00046

NF[25] 182.0 191.0 200.0 191.5 4.79283 0.09513 2 2539 0.35894 1.00067

NF[26] 174.0 186.0 195.0 185.7 5.44300 0.09932 1.8 3003 0.30464 1.00079

II. JAGS Code used to produce the model used to estimate right whale abundances from capture histories including recovery data

###### Made to run in r package runjags #####

#--------------------------------------

# Parameters:

# phi: survival probability

# gamma: removal entry probability

# p: capture probability

#--------------------------------------

# States (S):

# 1 not yet entered

# 2 alive

# 3 dead

# Observations (O):

# 1 seen

# 2 not seen

#--------------------------------------

model {

epsilon ~ dunif(0.01, 10) ### prior on standard deviation of catchability original ..... dunif(0.0001,4)

omega<- 1/(epsilon*epsilon) ### precision for use in jags/bugs

for (i in 1:(M))

{

Gotcha[i]~dnorm(0,omega) ### prior on random catchability of individuals

}

# Priors and constraints

sigma~dunif(0.001,10) ####### prior for sd of random year effect on phi

tau<-1/(sigma*sigma)

#### for pcap, Male becomes the intercept and is the value sex at t=0 or 1990

pie~dbeta(5,5) # prior for sex

for (u in 1:2) {

AlphaSex[u]~ dunif(-10, 10) # Prior for intecepts rate

}

AlphaTime[1]<-0

AlphaTime[2]<-0

for (t in 3:(n.occasions-1)) {

AlphaTime[t]~dunif(-10, 10)

}

# for survival parameters

for (t in 1:(n.occasions-1)){

gamma[t] ~ dunif(0, 1) # Prior for entry probabilities

} #t

eta[1]<-0 #### can only have entry at step 2, so ps[1,i,1,x] does not depend on phi

for (t in 2:(n.occasions-1)){

eta[t]~dnorm(0,tau)

} #t

BetaSex[1] ~ dunif(-10, 10) # Priors for sex effects on survival

BetaSex[2] ~ dunif(-10, 10)

BetaAge ~ dunif(-10, 10)

######### Probability models

for (i in 1:M){

sex[i]~dbern(pie)

for (t in 1:(n.occasions-1)){

logit(phi[i,t]) <- BetaSex[1] + BetaAge*Age[i,t] + BetaSex[2]*(sex[i])*Adult[i,t] + eta[t]

logit(pcap[i,t])<- AlphaSex[1] + AlphaSex[2]*(sex[i]) + AlphaTime[t] + Gotcha[i]

} #t

} #i

# Define state-transition and observation matrices

for (i in 1:M){

# Define probabilities of state S(t+1) given S(t)

for (t in 1:(n.occasions-1)){

ps[1,i,t,1] <- 1-gamma[t]

ps[1,i,t,2] <- gamma[t]

ps[1,i,t,3] <- 0

ps[2,i,t,1] <- 0

ps[2,i,t,2] <- phi[i,t]

ps[2,i,t,3] <- 1-phi[i,t]

ps[3,i,t,1] <- 0

ps[3,i,t,2] <- 0

ps[3,i,t,3] <- 1

# Define probabilities of O(t) given S(t)

po[1,i,t,1] <- 0

po[1,i,t,2] <- 1

po[2,i,t,1] <- pcap[i,t]

po[2,i,t,2] <- 1-pcap[i,t]

po[3,i,t,1] <- 0

po[3,i,t,2] <- 1

} #t

} #i

# for logistic parameters

for (t in 2:(n.occasions-1)){

pcap1[t-1] <- 1 / (1+exp(-AlphaSex[1]- AlphaSex[2]- AlphaTime[t])) # Back-transformed recapture of females

pcap2[t-1] <- 1 / (1+exp(-AlphaSex[1] - AlphaTime[t])) # Back-transformed recapture of males

phi0[t-1] <- 1 / (1+exp(-BetaSex[1]-eta[t])) # Back-transformed survival of calves

phi1[t-1] <- 1 / (1+exp(-BetaSex[1]-BetaAge*1-eta[t])) # Back-transformed survival of yearlings

phi2[t-1] <- 1 / (1+exp(-BetaSex[1]-BetaAge*2-eta[t])) # Back-transformed survival of 2-year-olds

phi3[t-1] <- 1 / (1+exp(-BetaSex[1]-BetaAge*3-eta[t])) # Back-transformed survival of 3-year-olds

phi4[t-1] <- 1 / (1+exp(-BetaSex[1]-BetaAge*4-eta[t])) # Back-transformed survival of 4-year-olds

phiaf[t-1] <- 1 / (1+exp(-BetaSex[1]-BetaSex[2]-BetaAge*5-eta[t])) # Back-transformed survival of adult females

phiam[t-1] <- 1 / (1+exp(-BetaSex[1]-BetaAge*5-eta[t])) # Back-transformed survival of adult males

}

# Likelihood

for (i in 1:M){

# Define latent state at first occasion ... in BPA this is always 1, but for RIWH I we have prior data about any individuals

z[i,1] <- 1 # Make sure that all M individuals are in state 1 at t=1

for (t in 2:n.occasions){

# State process: draw S(t) given S(t-1)

z[i,t] ~ dcat(ps[z[i,t-1], i, t-1,])

# Observation process: draw O(t) given S(t)

y[i,t] ~ dcat(po[z[i,t], i, t-1,])

} #t

} #i

# Calculate derived population parameters

for (t in 1:(n.occasions-1)){

qgamma[t] <- 1-gamma[t]

}

cprob[1] <- gamma[1] ###### BPA parameterization

for (t in 2:(n.occasions-1)){

cprob[t] <- gamma[t] * prod(qgamma[1:(t-1)])

} #t

psi <- sum(cprob[]) # Inclusion probability

for (t in 1:(n.occasions-1)){

b[t] <- cprob[t] / psi # Entry probability

} #t

for (i in 1:M){

for (t in 2:n.occasions){

al[i,t-1] <- equals(z[i,t], 2)

alm[i,t-1]<- al[i,t-1]*sex[i]

alf[i,t-1]<- al[i,t-1]*(1-sex[i])

# al[i,t-1] <- ifelse(z[i,t]=2,1,0)

} #t

for (t in 1:(n.occasions-1)){

d[i,t] <- equals(z[i,t]-al[i,t],0)

} #t

alive[i] <- sum(al[i,])

} #i

for (t in 1:(n.occasions-1)){

N[t] <- sum(al[,t]) # Actual population size

NF[t] <- sum(alf[,t])

NM[t] <- sum(alm[,t])

B[t] <- sum(d[,t]) # Number of entries

} ### t

for (i in 1:M){

w[i] <- 1-equals(alive[i],0)

} ### i

}

III. Simulations

Logit models are the primary form used in hierarchical Bayesian models across many disciplines when process or observation response variables (outcomes) are binary (see Gelman and Hill 2006 for a review). However, several aspects of our problem structure warrant some simulation testing against capture histories from known (i.e. synthetic) populations to verify that our computer code and the software used perform as desired. Hence, we created synthetic whale populations to which we applied known survival, birth and observation processes, estimated demographic parameters from the capture histories using our approach and compared the estimated values to known parameters. We examined the following questions:

1. Is including a random effect to model variation in mean survival rate among years adequate structure to detect a modest fixed change in mean survival?
2. As is a common problem in the generic Jolly-Seber mode, does individual capture heterogeneity result in a large bias in the estimation of N despite the attempt to accommodate this variance in the model?
3. Are model estimates sensitive to the assumption that animals of unknown age are included with the 5+ class?

To create our synthetic population and capture histories, we fixed the initial population size at 500 to which we added 40 calves after each of 10 survival intervals (11 capture occasions). The survival rates for ages 0-5+ were fixed on occasions 1-4 and 8-10 at 0.84, 0.88, 0.91, 0.94, 0.96, 0.97 respectively. For occasions 5-7, we reduced survival of the 6 age classes to 0.80, 0.85, 0.89, 0.92, 0.94, 0.96. The spread of survival rates was roughly equivalent to assuming that the logit(survival) increased linearly at a rate of 0.26/age class until 5 and remained constant for ages > 4. The age structure of the initial population of 500 animals was determined stochastically by assuming 40 calves born in each of 4 years prior to the start must become *a_i_* animals of an age class*_i_* as the result of a binomial trial N=40 and p= product of survival rates for the interval of their lives. For example, the number of 4 year olds would be the result of a binomial(n=40, p=0.84*0.88*0.91*0.96) trial. To speed calculations by reducing the number of parameters, sexes of simulated populations were assumed to share the same age-specific sex survival rates.

To produce a realized data set of capture histories and their associated derived known states from the true synthetic population just described, we applied constant mean periodic recapture probability of mu=0.72. For each sampling occasion during which the i^th^ individual was alive, that individuals was captured at a rate of *p,*  where logit(*p*) = 0.95 + normal(0, 1.5), which leads to highly skewed individual capture heterogeneity (Figure III.a). As with the right whale data, the values in the known state matrix for the synthetic capture histories were, NA= unknown, 1=not entered, 2=alive, and 3=dead. States at all occasions between the first and last captures were classed as 2. Animals first caught as calves were assigned known states =1 prior to their first capture. Animals not first captured as calves had their known state set to 2 the occasion prior to their first capture as well as those occasions up until their last live sighting or recovery. We assumed a recovery rate of 0.3 so that all animals that died during and were recovered during the study period had all states classified to 3 following their last capture. All other states were set to NA.

Figure III.a Example distribution of individual

capture probabilities in simulation trials.


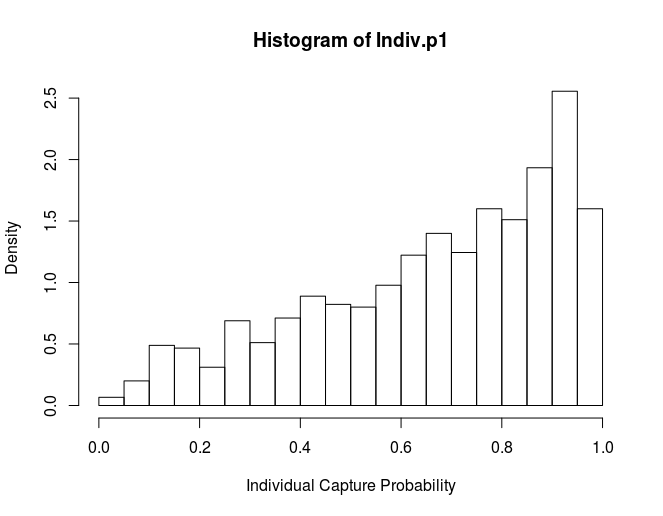


To examine the sensitivity of the right whale model to setting unknown ages in those data to 5, we ran two versions of each synthetic population. The control used the actual age matrix from the generated population. The second generated a new age-at-occasion matrix in which any individual not captured the first time at age class 0 (a calf) had all ages-at-occasion set to 5. Hierarchical models were run on the synthetic data similarly to the right whale data except the augmentation sample size was set to 250 and the final MCMC chain sample was 10,000.

Results Summary. In simulated trials the main model (simulations excluded sex effect) produced nearly unbiased estimates of true abundance (Figure III.b) despite having average annual capture probabilities lower than were estimated for our right whale data. There was a tendency in the simulations to under estimate abundance in the first two occasion and over estimate for the last occasion, with the latter being slight (Figure III.c). However, simulated known states were not enhance by information gained after the last occasion as with the right whale data. As expected, pooling unknown ages into the 5+ class tended to bias high survival of the calf age class, but due to the numerical dominance of the 5+ class, estimated survival of that age class was always close to the true survival (Figure III.d).

Figure III.b. Overall distribution of bias in estimates of abundances among simulation trials.


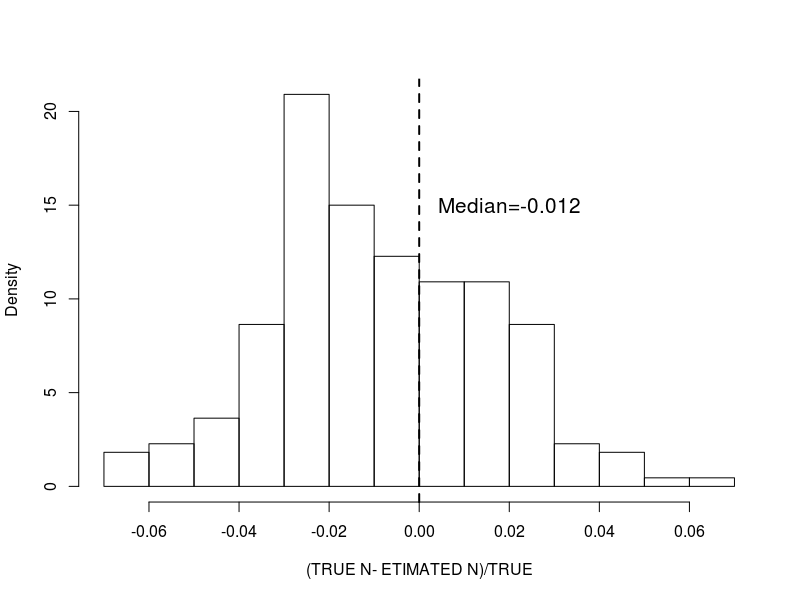


Figure III.c. Box plots of estimated abundances by occasion from simulation trials.


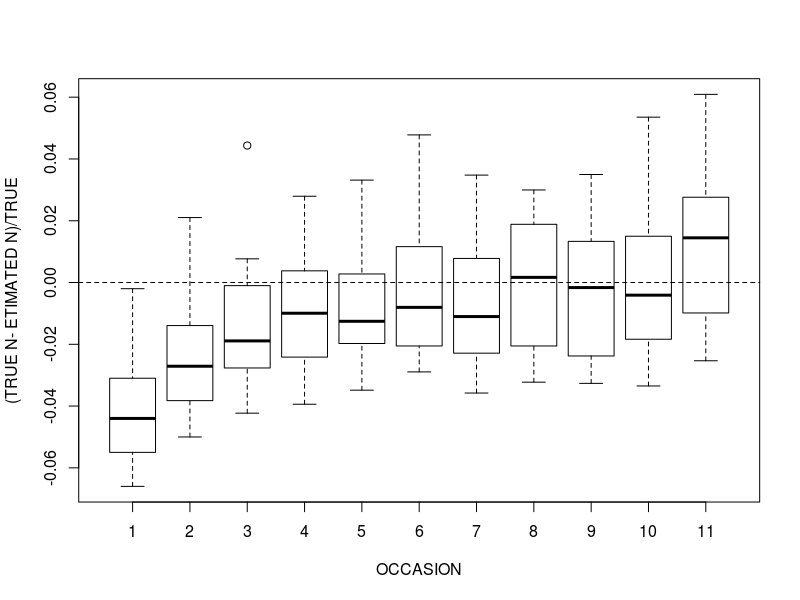
Figure III.d. Example estimated age-specific (age classes 0 and 5+ only) verses true survival in time-constant survival case (Horizontal lines are True with class= 0 below class=5+)


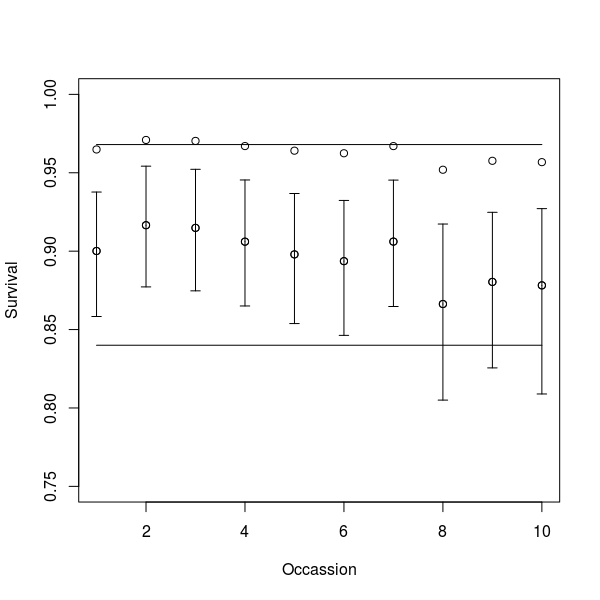


References

Gelman A. and Hill J. 2006. data analysis using regression and multilevel/hierarchical models. Cambridge University Press.
